# Metabolic characteristics related to potentially toxic elements in the blood of young adults in China: a cross-sectional study

**DOI:** 10.3389/fnut.2025.1678706

**Published:** 2025-11-19

**Authors:** Lei Zhang, Wen Xu, Mingyu Feng, Jia Zhang, Zhenzhong Wang, Wenfeng Kang, Yi Liu, Beibei Yang, Yuming Guo, Peng Lu

**Affiliations:** 1 School of Public Health, Binzhou Medical University, Yantai, Shandong, China; 2 Department of Traditional Rehabilitation, School of Special Education and Rehabilitation, Binzhou Medical University, Yantai, China; 3 Yantai Affiliated Hospital of Binzhou Medical University, Yantai, Shandong, China; 4 Department of Epidemiology and Preventive Medicine, School of Public Health and Preventive Medicine, Monash University, Melbourne, VIC, Australia

**Keywords:** metabolomics, toxic metals, nicotinate and nicotinamide metabolism, vitamin B6 metabolism, young adults

## Abstract

Epidemiological evidence links essential and potentially toxic metals exposure to impaired lung function, but underlying biological mechanisms remain unclear. Building on previous findings demonstrating impaired lung function from copper (Cu), cerium (Ce), and iron (Fe) exposure, this study explored associated blood metabolomic signatures in young adults. 1,742 first-year university students enrolled in 2019 were included in this study in Shandong Province, China. Whole blood metal concentrations (ICP-MS) and metabolomic profiles (LC–MS) were assessed. Metals were classified into quartiles and analyzed using ANOVA analysis and multiple linear regression to assess their association with metabolites. Metabolomic analysis identified Cu exposure significantly associated with 45 metabolites across 14 pathways. Ce exposure linked to 25 metabolites enriched in seven pathways. Fe exposure associated with 26 metabolites in 13 pathways. All three metals both dysregulated nicotinate and nicotinamide metabolism and vitamin B6 metabolism. Additionally, Cu and Ce disrupted unsaturated fatty acid biosynthesis. These findings reveal that specific metabolic pathways—particularly those involving nicotinamide and vitamin B6—may serve as potential nutritional intervention targets for mitigating metal-induced lung function impairment in young adults.

## 1 Introduction

The relationship between potentially toxic elements (PTEs) and human health has garnered increasing scientific attention, particularly regarding respiratory outcomes (1). Metals such as iron, copper, and cerium, while essential in trace amounts, demonstrate complex toxicological profiles when exposure exceeds physiological thresholds. Emerging evidence suggests these metals may impair pulmonary function through multiple mechanisms, including oxidative stress induction [evidenced by elevated biomarkers of oxidative damage (2), inflammatory cytokine upregulation (3), and disruption of critical metabolic pathways (4)]. Notably, cerium oxide nanoparticles have been specifically linked to lung fibrosis (5), while copper/iron-mediated Fenton reactions contribute to cellular damage (6). Our previous research has further established associations between impaired lung function and exposure to multiple PTEs, identifying Cu and cerium (Ce) as detrimental elements while recognizing iron (Fe) as potentially protective (7). These findings underscore the dual nature of essential metals as both nutrients and potential toxicants depending on exposure contexts.

Metabolomics captures dynamic metabolic alterations and offers mechanistic insights into the health impacts of environmental exposures (8). This approach has gained prominence in elucidating the biological pathways underlying metal toxicity (9). For instance, an epidemiological study involving 186 steel factory workers in Anhui, China, found that multi-metal exposure might disrupt lipid and amino acid metabolism, particularly glycerophospholipid metabolism and the arginine and proline metabolism pathways (10). Correspondingly, a study of 456 residents in a South Korean mining area revealed various metal-associated metabolites involved in glycolysis and amino acid metabolism pathways (11). However, research on metabolic pathways and potential mechanisms associated with PTEs exposure in large-scale young populations remains lacking.

Based on the above evidence, we conducted this cross-sectional study to investigate the association between exposure to Cu, Ce, and Fe and blood metabolomic profiles in young adults, with the goal of elucidating the biological mechanisms underlying PTEs-induced adverse health effects and to provide evidence for nutritional intervention strategies.

## 2 Methods

### 2.1 Study population

Participants in this cross-sectional study were drawn from the Chinese undergraduate cohort (CUC). The primary goal of this cohort is to explore the association between environmental exposure and health outcomes in Shandong province, China (12, 13). A total of 2,733 freshmen at Binzhou Medical University were recruited between September 2 and September 10, 2019. The inclusion criteria were defined as follows: (1) enrolled in 2019; (2) age ≥18 years; (3) documented residency in Shandong Province prior to 2019; (4) not menstruating at the time; (5) without clinically diagnosed metabolic disorders. After enrollment, participants were requested to fill out questionnaires (including basic information, lifestyle, dietary situation, environmental factors, sleep conditions, and disease status, see Supplementary material for details) and collect blood samples. We excluded individuals with incomplete questionnaires, missing blood samples, or residence outside Shandong Province, resulting in 1,742 eligible participants for final analysis. Informed consent was obtained from each participant. This study was approved by the Binzhou Medical University ethics committee (No. 2019075). Supplementary Figure S1 illustrates the flowchart of the participant enrollment process.

### 2.2 Blood collection and measurement of metal concentrations

Venous blood samples (10 mL) were collected from fasted participants using EDTA-coated vacutainers between September 2 and 10, 2019, following an 8-h fasting period. At room temperature, 0.35 mL of whole blood samples were aliquoted for subsequent metal detection. The remaining blood samples were centrifuged at 3,500 rpm for 10 min to obtain plasma for untargeted metabolomics analysis. All samples were then transferred and stored at −80 °C. Details on the sample collection and measurement process can be found in the Supplementary material.

### 2.3 Metabolomics and lipidomics analysis

The extraction of metabolites and lipids from plasma samples was conducted using the liquid–liquid extraction method. Following the previous procedure (14), 100 μL of plasma was mixed with four volumes of cold chloroform and methanol (2:1) in a 1.5 mL polypropylene EP tube for extraction. Then the mixture was spun at 13,000 rpm for 15 min, separating into upper and lower phases. The phases were vacuum-evaporated in a quartz tube to remove the solvent CVE-3100 Vacuum Centrifugal Concentrator (Tokyo Rika Co., Ltd.) under conditions of 4 °C, 4,000 rpm, and 50 Pa. Subsequently, the desiccated samples were stored at −80 °C for later analysis by liquid chromatography-mass spectrometry (LC–MS). Metabolomics and lipidomics were performed on an Ultimate 3000 UHPLC system coupled with Q-Exactive HF MS (Thermo Fisher Scientific, Waltham, MA, USA) as previously described. For the metabolomics, an Xbridge amide column (100 × 2.1 mm i.d., 3.5 um; Waters, USA) was used at 30 °C. For the lipidomics, a reversed-phase BEH C_18_ column (2.1 mm × 100 mm, 2.5 μm, Waters, USA) was used at 40 °C. Detailed methodology described in the Supplementary material.

### 2.4 Statistical analysis

Characteristics of the study participants were presented as mean ± standard deviation (SD) for continuous variables and as frequency (percentages) for categorical variables. And the whole blood concentrations of metal were reported as median (interquartile range, IQR). Based on our previous study, this study focused on three essential and potentially toxic metals (Cu, Ce, and Fe). Both whole blood metal concentrations and serum metabolites were log10 transformed prior to further analyses. Based on our previous study (15), two approaches were employed to identify exposure-related differential metabolites using a threshold of *p* < 0.05. Firstly, one-way analysis of variance (ANOVA) and *post-hoc* Duncan analysis were employed to assess metabolic changes associated with blood Cu, Fe, and Ce exposure. Secondly, the multivariable linear regression models were used to investigate the associations of blood Cu, Fe, and Ce levels and metabolite alterations. To adjust for potential confounding, we included covariates in our model. Specifically, the covariates consisted of age, sex, body mass index (BMI), socio-economic status (advantaged or disadvantaged), residence (urban or rural), disease history, smoking, passive smoking, and drinking history. The socioeconomic level was classified into advantaged and disadvantaged groups based on an annual family income threshold of 50,000 RMB.

The identified metabolites were cross-referenced with the Human Metabolome Database (HDMB) and Kyoto Encyclopedia of Genes and Genomes (KEGG) databases. Pathway analysis was conducted among significant metabolites associated with each metal using MetaboAnalyst 6.0 (16). Finally, metabolic pathways with both the raw *p*-value or the *FDR*-adjusted *p*-value < 0.01 were selected for subsequent analysis.

All statistical analyses were performed in R (version 4.2.1). And a *p* value < 0.05 was considered statistically significant.

## 3 Results

### 3.1 Demographic characteristics of study participants

Table 1 summarizes the characteristics of the study population (*n* = 1,742), which included 1,002 males (57.52%) and 740 females (42.48%). The mean age of participants was 19.38 ± 0.64 years. Among them, 50.23% were urban residents. Additionally, 40.41% reported an annual household income below 50,000 RMB (≈7,730 USD). A total of 25 participants reported current smoking, and 89 reported alcohol consumption. Additionally, 213 participants had a history of smoking. The median concentrations of blood metals were as follows: copper (Cu), 852.93 (765.41, 953.63) ng/mL; cerium (Ce), 0.11 (0.08, 0.15) ng/mL; and iron (Fe), 500,081.92 (433,110.80, 558,129.93) ng/mL. Descriptive statistics for blood metal levels in different gender groups were presented in Supplementary Table S1.

**Table 1:** Demographic characteristics of all participants.

Characteristic	All participants
Age (years)	19.38 ± 0.64
BMI (kg/m^2^)	22.13 ± 3.89
Sex (%)	
Male	740 (42.48)
Female	1,002 (57.52)
Registered residence (%)	
Urban	875 (50.23)
Rural	867 (49.77)
Socioeconomic status (%)	
Disadvantage (< US$7,730)	704 (40.41)
Advantage (≥ US$7,730)	1,038 (59.59)
Cigarette smoke exposure (%)	
Active smoking	25 (1.44)
Passive smoking	213 (12.22)
Alcohol consumption (%)	
Yes	580 (33.30)
No	1,162 (66.70)
Metal concentrations (ng/mL)	
Cu	852.93 (765.41, 953.63)
Fe	500,081.92 (433,110.80, 558,129.93)
Ce	0.109 (0.08, 0.15)

### 3.2 Altered metabolites and metabolic pathways associated with Cu

**Figure 1:**
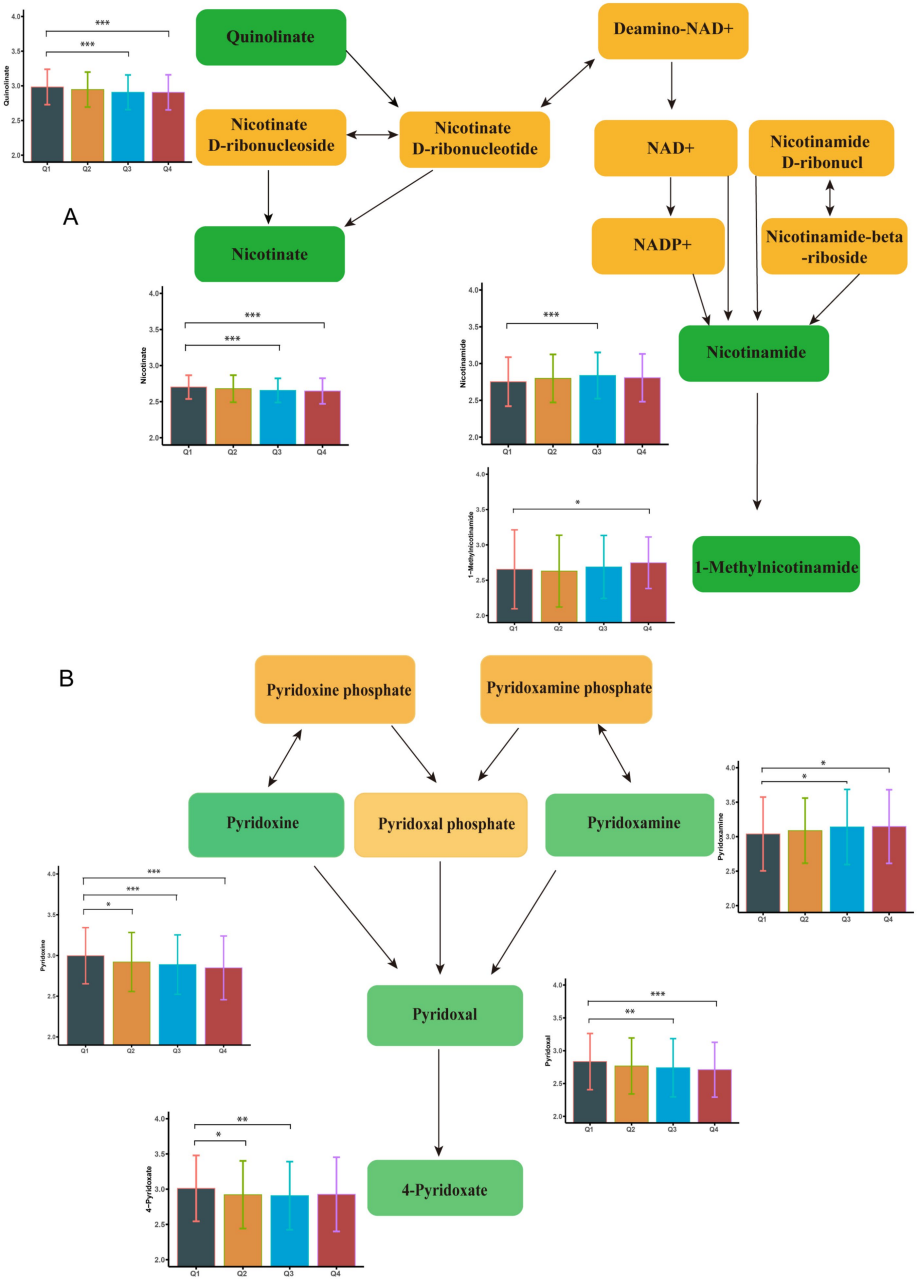
Metabolome changes associated with blood copper levels. **(A)** Vitamin B6 metabolic pathway. **(B)** Niacin and Niacinamide metabolic pathways. Q1: 25th percentile; Q2: 50th percentile; Q3: 75th percentile; Q4: 90th percentile; * represents *p* < 0.05, ** represents *p* < 0.01, ***represents *p* < 0.001, compared to the Q1 group.

**Figure 2:**
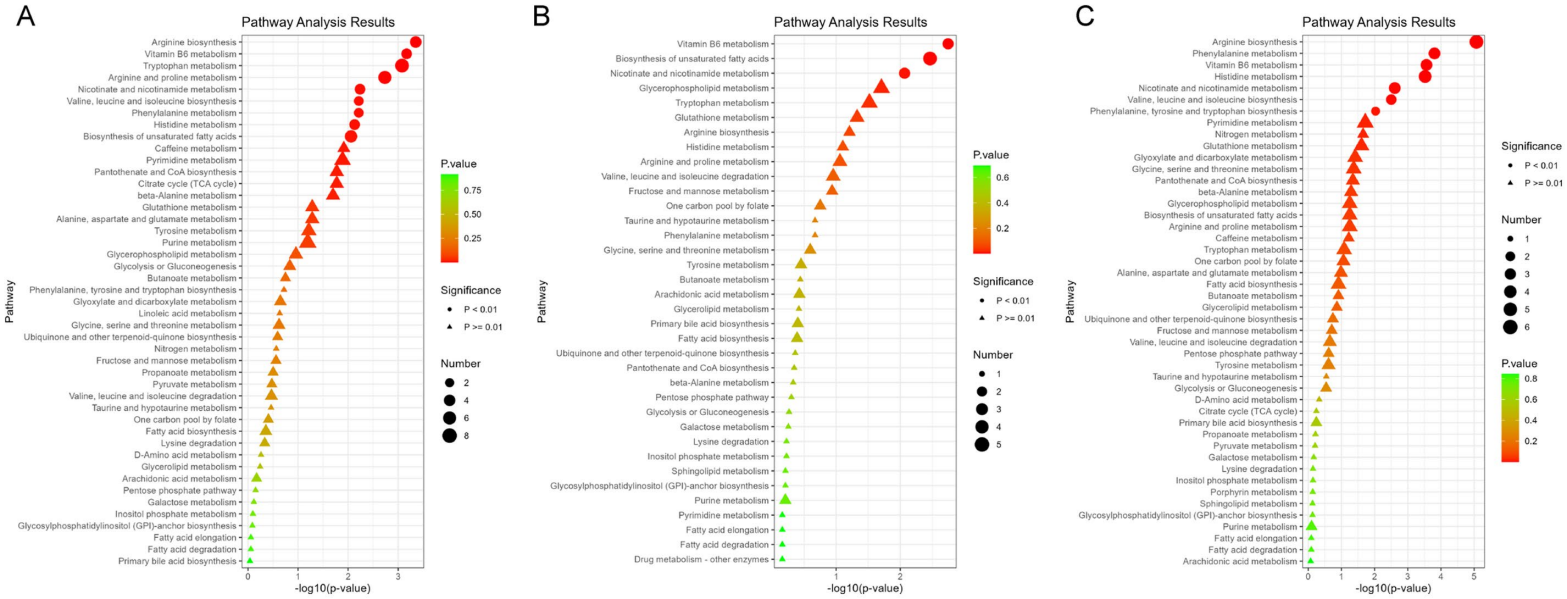
Enrichment analysis of metabolic pathways associated with potentially toxic elements. **(A)** Enrichment results of Ce metabolic pathway. **(B)** Enrichment results of Cu metabolic pathway. **(C)** Enrichment results of Fe metabolic pathway. ● indicate metabolic pathways with *p* < 0.01, while ▲ represent metabolic pathways with *p* > 0.01.

Following a two-step statistical analysis, a total of 271 metabolites exhibited significant correlations with increasing blood Cu concentrations (*p* < 0.05) (Supplementary Table S2). Among them, 36 metabolites (*p* < 0.01) were further enriched in nine metabolic pathways. The following metabolites showed a negative correlation with blood copper levels: Arginine, Ornithine, Pyridoxine, Pyridoxal, 4-Pyridoxate, Leucine, 4-Methyl-2-oxopentanoate. In contrast, the expression of Nicotinamide, Pyridoxamine, and 1-Methylnicotinamide was positively associated with blood Cu levels. Additional Cu-related metabolites are presented in Supplementary Figures S2–S4 and Supplementary Table S3. Key altered pathways included: arginine biosynthesis, vitamin B6 metabolism, tryptophan metabolism, arginine and proline metabolism, nicotinate and nicotinamide metabolism, valine, leucine and isoleucine biosynthesis, phenylalanine metabolism, histidine metabolism, and biosynthesis of unsaturated fatty acids (*p* < 0.01). Figure 1 illustrates the metabolites enriched in the pathways of nicotinate and nicotinamide metabolism and vitamin B6 metabolism following Cu exposure. Other significantly changed metabolic pathways associated with Cu was shown in Figure 2A.

### 3.3 Metabolites and metabolic pathways associated with Ce

**Figure 3:**
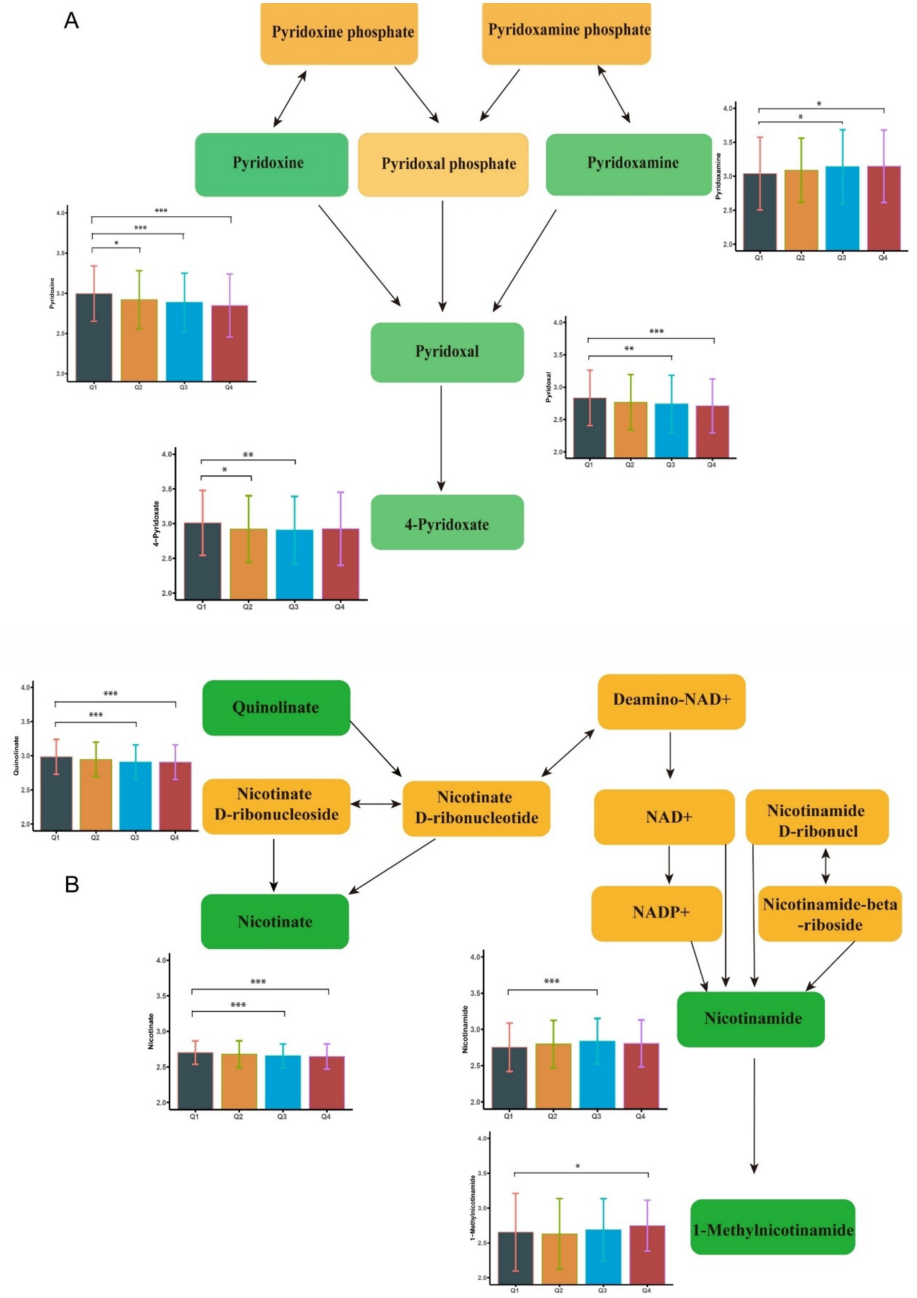
Metabolome changes associated with blood iron levels. **(A)** Vitamin B6 metabolic pathway. **(B)** Niacin and Niacinamide metabolic pathways. Q1: 25th percentile; Q2: 50th percentile; Q3: 75th percentile; Q4: 90th percentile; * represents *p* < 0.05, ** represents *p* < 0.01, *** represents *p* < 0.001, compared to the Q1 group.

A total of 179 differential metabolites were linked to increased blood Ce concentrations (*p* < 0.05). Of these, 36 metabolites showed statistically significant changes (*p* < 0.01) (Supplementary Table S4). Nine metabolites were found to be enriched in three metabolic pathways (*p* < 0.01). Specifically, the levels of Pyridoxal, 4-Pyridoxate, 1-Methylnicotinamide, and Quinolinate were negatively associated with increased blood Ce concentrations. In contrast, the levels of Nicotinate and (6Z,9Z,12Z)-Octadecatrienoic acid were positively associated with elevated Ce levels. Representative altered metabolites are shown in Figure 3. These metabolites were enriched in three key pathways: vitamin B6 metabolism, biosynthesis of unsaturated fatty acids, and nicotinate and nicotinamide metabolism. Additional Ce-related metabolites are provided in Supplementary Figure S5 and Supplementary Table S3. Additional enriched pathways are illustrated in Figure 2B.

### 3.4 Metabolites and metabolic pathways associated with Fe

**Figure 4:**
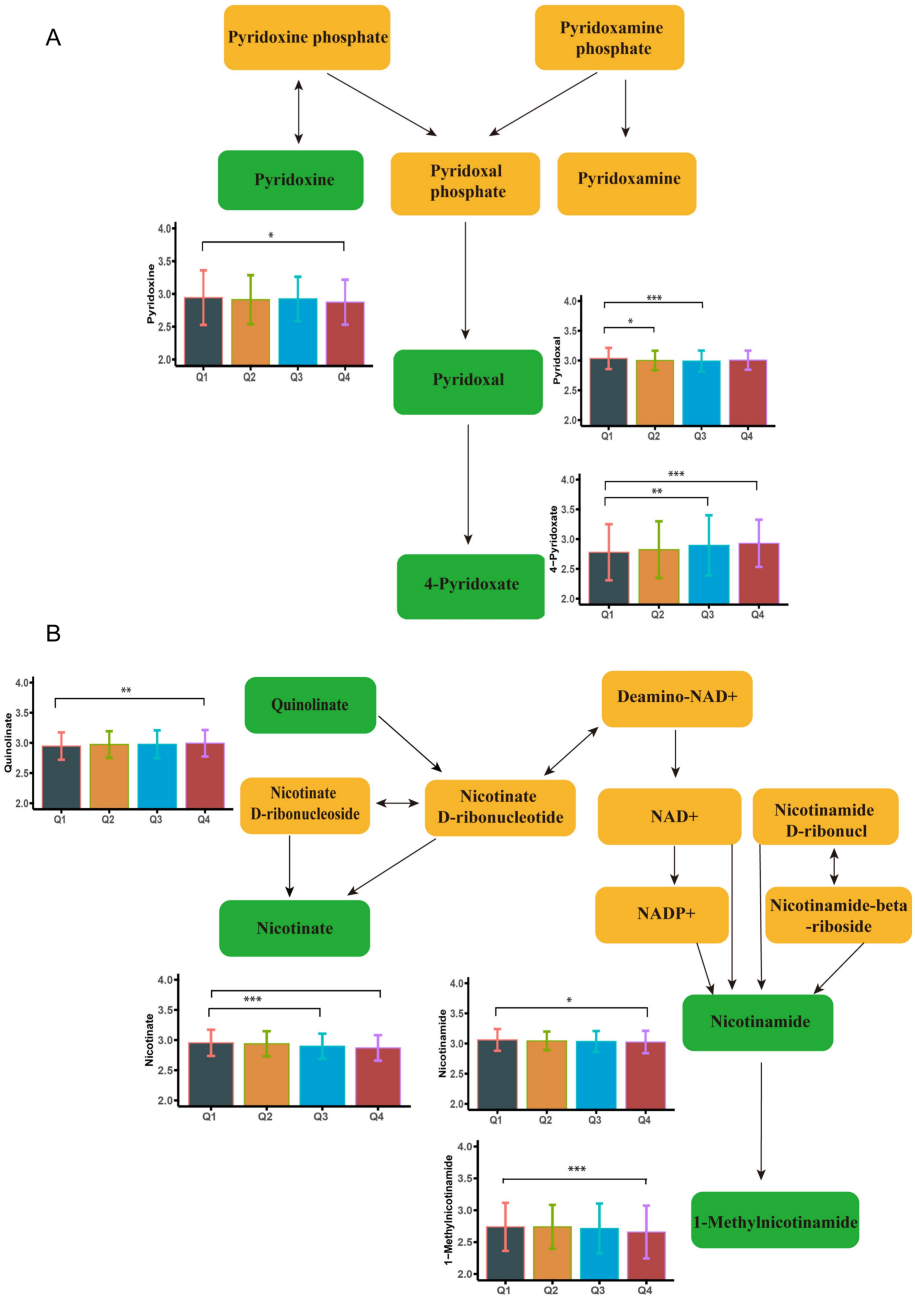
Metabolome changes associated with blood cerium levels. **(A)** Niacin and Niacinamide metabolic pathways. **(B)** Vitamin B6 metabolic pathway. Q1: 25th percentile; Q2: 50th percentile; Q3: 75th percentile; Q4: 90th percentile; * represents *p* < 0.05, ** represents *p* < 0.01, *** represents *p* < 0.001, compared to the Q1 group.

There were 271 differential metabolites associated with elevated blood Fe levels (*P*<0.05). Among these, 18 metabolites were significantly enriched in seven metabolic pathways (Supplementary Table S5). As shown in Figure 4, nicotinate levels were upregulated, whereas 1-methylnicotinamide, pyridoxal, and 4-pyridoxate levels were downregulated with increasing Fe concentrations. These metabolites were enriched in the nicotinate and nicotinamide metabolism pathway and the vitamin B6 metabolism pathway. Figure 2C shows other significantly altered metabolic pathways. Additional significantly altered metabolites associated with Fe are presented in Supplementary Figures S6, S7 and Supplementary Table S3. A sensitivity analysis excluding participants with a history of smoking was conducted, and the results remained consistent (Supplementary Tables S6–S8).

## 4 Discussion

This study estimated the associations between blood Cu, Ce, and Fe concentrations and the plasma metabolome among Chinese young adults. Notably, the commonly dysregulated pathways included nicotinate and nicotinamide metabolism and vitamin B6 metabolism. Furthermore, the biosynthesis of unsaturated fatty acids showed dysregulated by both Cu and Ce, while arginine biosynthesis and valine, leucine, and isoleucine biosynthesis were affected by both Cu and Fe. These identified biological pathways were closely linked to oxidative stress and inflammatory responses.

Blood metal concentrations are relatively stable indicators of metal exposure (17). In a Chinese study of 648 participants aged 12–60 years, the geometric mean of blood Cu concentration was 802.40 (95% CI: 787–820) ng/mL (18). Our findings demonstrate comparable blood copper levels (855.83 (95% CI: 843.98–864.62) ng/mL) to these previous reports. A Spanish cross-sectional study reported that the median Ce concentration was 0.16 ng/mL in men and 0.15 ng/mL in women (19), while a Norwegian study of 1,011 adults (aged 20–91 years) reported that whole blood Fe concentration was 541,000 ± 133,000 ng/mL. These values are consistent with those observed in our study (0.16 ± 1.27 ng/mL for Ce and 492,712 ± 95,391.75 ng/mL for Fe). Although we did not obtain data on iron supplementation, anaemia, inflammatory status, or dietary intake for our study subjects, these factors have been highlighted in previous research as having a significant impact on blood iron levels or serum ferritin. It was reported that supplementing iron at lunchtime for eight consecutive weeks increased serum ferritin levels by 10.7 μg/L (20). In anaemic children, adolescents, and young adults, iron supplementation (>30 mg/day) markedly elevates haemoglobin levels (increase up to 10.24 g/L), with more pronounced effects observed during short-term interventions (21). Notably, intravenous iron administration under inflammatory conditions elevates oxidative stress and inflammatory markers (22). Ce serves as a crucial component in automotive exhaust purification catalysts, with potential exposure occurring during production and recycling processes (23). Meanwhile, as essential elements, abnormal blood levels of Fe and Cu might relate to dietary imbalances. For instance, zinc deficiency (below 6,017 μg/L whole blood) frequently accompanies disturbances in Fe and Cu metabolism (24). Disparities in Cu and selenium intake might also influence their respective blood concentrations (25).

Significant associations were identified between whole blood Cu and Ce concentrations and the vitamin B6 metabolism pathway, resulting in distinct regulatory patterns of associated metabolites. Similar associations were also observed for Fe. Specifically, Cu exposure was found to downregulate pyridoxine and pyridoxal levels. Ce exposure downregulated pyridoxal and 4-pyridoxate, whereas Fe exposure upregulated 4-pyridoxate levels. It should be noted that pyridoxine and pyridoxal represent two biologically active forms of vitamin B6 (26), whereas 4-pyridoxate is a key metabolic derivative of vitamin B6 (27). As an established antioxidant, vitamin B6 serves as an essential coenzyme in the glutathione-mediated antioxidant defense system (42). Although epidemiological investigations examining the relationship between vitamin B6 metabolism and pulmonary function remain scarce, a previous cross-sectional study reported a significant positive correlation between dietary vitamin B6 intake and both FEV_1_ and FVC (27). The combination of vitamin B6 and branched-chain amino acids (BCAAs) activates the PI3K/AKT/mTOR pathway, improving muscle and intestinal morphology, thereby indirectly supporting respiratory muscle function (28). The combined findings suggested that vitamin B6 may represent a promising nutritional intervention target for Cu, Fe, and Ce exposure-induced pulmonary health impairments, primarily involving inflammatory responses and oxidative stress mechanisms.

The metabolism of niacin and niacinamide represents another consistently disturbed pathway. Cu exposure was found to reduce nicotinate metabolite levels, whereas Fe induced quinolinate upregulation. In addition, Ce downregulated 1-methylnicotinamide (1-MNA) levels while upregulating nicotinate. Niacinamide modulates the NAD^+^/NADH ratio to inhibit nuclear factor κB (NF-κB) signaling, exerting anti-inflammatory effects (29, 30). However, high doses of nicotinate or 1-MNA may have adverse effects. Studies have shown that 1-MNA can induce oxidative stress and generate superoxide anions, contributing to the pathogenesis of Parkinson’s disease. High-dose intake of nicotinate has been shown to significantly reduce plasma vitamin B6 levels in mice. Quinolinate serves as a crucial metabolic precursor in the *de novo* NAD^+^ synthesis pathway (31). Importantly, studies have nicotinamide’s protective effects against acute lung injury through suppression of NF-κB-mediated pro-inflammatory mediator secretion (32). These collective findings suggest that Cu, Fe, and Ce exposure adversely affect lung function by disrupting the nicotinate and nicotinamide metabolic pathways, thereby potentiating inflammation and oxidative stress.

The dysregulation of unsaturated fatty acid biosynthesis further highlights the involvement of lipid metabolism in Cu and Ce exposure-related health effects. Emerging evidence indicates that unsaturated fatty acids, particularly polyunsaturated fatty acids (PUFAs), possess anti-inflammatory properties by modulating inflammatory mediator production. *In vivo* experimental also suggests that disruption of PUFA metabolic pathways may contribute to lung inflammation and tissue injury (33). In chronic obstructive pulmonary disease (COPD), circulating omega-3 PUFA levels may modify the relationship between long-term exposure to air pollutants and COPD risk (34, 35). PUFA supplements (such as fish oil) have demonstrated clear benefits in cardiovascular and metabolic diseases, but their application in pulmonary conditions (such as COPD and pulmonary fibrosis) requires further clinical evidence (36). This study found that blood Cu and Ce levels might interfere with the synthesis of PUFA, but we did not assess the relationship between PUFAs and lung function in young individuals. Although previous evidence suggested that supplementation with PUFAs may serve as an effective antagonist against the adverse health effects induced by Cu and Ce exposure, the clinical benefits require validation through controlled trials or clinical studies to substantiate these findings.

Our findings suggest that elevated exposure to Cu and Fe may disrupt the biosynthesis pathways of arginine, valine, leucine, and isoleucine. Arginine can effectively alleviate oxidative stress by activating the Nrf2–Keap1 pathway, promoting glutathione synthesis, and upregulating the expression of antioxidant enzymes. This observation aligns with a clinical study involving 32 healthy pregnant women, which demonstrated that Cu and Fe exposure significantly influenced amino acid metabolism, particularly arginine metabolism (37). Previous research demonstrated that arginine administration improved pulmonary function in a rat model of chronic obstructive pulmonary disease (COPD) by regulating the ROS/NLRP3/NF-κB signaling pathway and reducing inflammatory mediator levels (43). Similarly, leucine was found to significantly inhibit IL-8 production by suppressing NF-κB phosphorylation (38). These findings are further supported by clinical evidence showing significantly lower plasma leucine levels in COPD patients compared to healthy controls (39). These findings imply that Cu and Fe exposure may impair lung health by interfering with amino acid biosynthesis and promoting pulmonary inflammation. Disruption of these amino acid pathways may compromise antioxidant defense and immune modulation, exacerbating inflammatory processes in the lungs and potentially leading to long-term functional impairment.

This study has several notable strengths. Firstly, to the best of our knowledge, it is the first to examine the associations between blood Cu, Ce, and Fe concentrations and metabolic profiles in a substantial cohort of young adults. Secondly, we adjusted for a wide range of demographic, lifestyle, and socioeconomic factors, which helped reduce confounding and better isolate the true associations. Thirdly, the use of blood metal concentrations provides a more reliable measure of internal exposure compared to urinary biomarkers. Nevertheless, certain limitations should be acknowledged. Firstly, the analysis was restricted to single-pollutant models, which precluded examination of potential interactive or joint effects among multiple metals. Future large-scale studies should consider metal mixtures to better understand their combined impacts on the human metabolome. Secondly, owing to the cross-sectional study design, it is not possible to infer a causal relationship between metal exposure and metabolic alterations. Furthermore, the absence of a control group prevented comparison of the exposed group with an unexposed group possessing similar characteristics. Previous research had emphasised that the lack of a valid control group hinders researchers’ ability to determine whether observed associations stem from causation or confounding factors, potentially heightening concerns regarding bias risk (40, 41). Thirdly, some of the identified metabolites have not been previously validated, and further experimental confirmation is warranted. Finally, the absence of data on iron supplementation, anemia, inflammatory conditions, and dietary intake limits the interpretability of blood iron levels in relation to environmental exposures.

## 5 Conclusion

This cross-sectional study of 1,742 young adults in China revealed associations between exposure to Cu, Ce, and Fe and distinct alterations in blood metabolomic profiles. Notably, these metals dysregulated nicotinate, nicotinamide, and vitamin B6 metabolism, and Cu and Ce additionally disrupted unsaturated fatty acid biosynthesis. These metabolic disturbances were linked to known mechanisms of oxidative stress and inflammation. While the findings suggest that nutritional pathways involving vitamin B6 and nicotinamide may be important for understanding the adverse effects associated with metal exposure, further longitudinal and experimental studies are needed to confirm these mechanisms and assess the potential for dietary interventions.

## Data Availability

The datasets presented in this study can be found in online repositories. The names of the repository/repositories and accession number(s) can be found in the article/Supplementary material.
