# Supplementary material for: Metabolic characteristics related to potentially toxic elements in the blood of young adults in China: a cross-sectional study
Source: Front Nutr. 2025 Nov 19;12:1678706. doi: 10.3389/fnut.2025.1678706 (PMC12672240; doi:10.3389/fnut.2025.1678706)
Supplement: Supplementary file 1 [file Data_Sheet_1.PDF]

Research on Health Influencing Factors of Binzhou Medical University Students (First Survey)

Informed Consent Form

Dear student,

This questionnaire is part of an epidemiological study on environmental exposure and health effects. Since students come from different regions before entering the university, their exposure levels to environmental pollutants and the concentrations of pollutants in their bodies vary, leading to different potential impacts on health. Based on this fact, our research team sincerely invites you to participate in the study titled “Investigation on Internal Exposure to Environmental Pollutants”, which aims to explore how various pollutants may affect the human body. The results of this survey will be submitted to our research group for further analysis. Your personal information will not be disclosed to any third party under any circumstances. Participation in this study is completely voluntary, and you may withdraw at any time. If you choose to withdraw midway, the research team will stop collecting your information. [Single choice question]

*○ I have read the informed consent and agree to participate in this study

Name [Fill in the blank] *

____

Gender [Single choice] *

○Female ○Male

Basic Information：[Matrix text question] *

Ethnicity:	________________
Date of Birth:	________________
ID Number:	________________

Student ID:	____________________________
Contact Number:	____________________________
QQ Number:	____________________________
WeChat ID:	____________________________
Height (cm):	____________________________
Weight (kg):	____________________________
On average, how long do you use your mobile phone or tablet each day (in minutes)?	____________________________

Pre-enrollment Information

Home Address Before University Enrollment:__________________________ Street (Town/Village):_________________ [Fill-in question] *

Name of High School (the one you attended for the longest period):_________________ :

Address___________________________(Town/Village)_________________

Boarding at school:___ [Fill-in question] *

Name of Middle School (the one you attended for the longest period):_________________

Address___________________________(Town/Village)_________________

Boarding at school:___ [Fill-in question] *

Name of Primary School (the one you attended for the longest period):_________________

Address___________________________(Town/Village)_________________

Boarding at school:___ [Fill-in question] *

Home Address at Birth:________________________ (Town/Village)：

____

Current Number of Household Members:___

Total Household Income Last Year:________ [Fill-in question] *

Smoking Status

Do you smoke? (Smoking is defined as smoking at least one cigarette per day on average for six months or longer)： [Single Choice Question] *

○Smoking ○Former smoker

○Never or occasional smoker (averaging no more than one cigarette per day)

Smoking Status [Matrix Text Question] *

Age at Smoking Initiation:	________________________
Current Average Daily Cigarettes (pcs):	________________________

Dependent on (Question: Do you smoke? [Smoking is defined as averaging at least one cigarette per day for six months or more], first option)

Smoking Habits [Matrix Text Question]*

Age at Smoking	________________________

Initiation:	
Age at Smoking Cessation:	______________________
Average Daily Cigarettes Before Quitting (pcs):	______________________

Dependent on (Question: Do you smoke? [Smoking is defined as averaging at least one cigarette per day for six months or more], second option)

Have you been regularly exposed to secondhand smoke (inhaling smoke exhaled by others for an average of 15 minutes or more per day)? [Single Choice Question] *

○No or rarely ○Yes

Dependent on (Question: Do you smoke? [Smoking is defined as averaging at least one cigarette per day for six months or more], third option)

Average Daily Secondhand Smoke Exposure: ___ hours ___ minutes

Duration of Secondhand Smoke Exposure: ___ years [Fill-in-the-blank Question] *

Dependent on (Question: Have you been regularly exposed to secondhand smoke [inhaling smoke exhaled by others for an average of 15 minutes or more per day], second option)

Alcohol Consumption

Do you drink alcohol (averaging at least 12 times per year over the past three years)? [Single Choice Question] *

○Current drinker ○Former drinker

○Never or occasional drinker

Alcohol Consumption [Matrix Text Question] *

Age at Drinking Initiation:	________________
Age at Alcohol Cessation:	________________

Dependent on (Question: Do you drink alcohol [averaging at least 12 times per year over the past three years], second option)

What types of alcohol do you usually drink? (Multiple Choice) [Multiple Choice Question]*

□Beer □Baijiu

□Red Wine / Wine □Huangjiu / Rice Wine

□Other ________________*

Dependent on (Question: Do you drink alcohol [averaging at least 12 times per year over the past three years], first option)

What types of alcohol did you usually drink before quitting? (Multiple Choice) [Multiple Choice Question] * *

□Beer □Baijiu

□Red Wine / Wine □Huangjiu / Rice Wine

□Other ________________*

Dependent on (Question: Do you drink alcohol [averaging at least 12 times per year over the past three years], first option)

Beer Drinking Frequency: ____ times/month, ____ bottles/time. [Fill-in-the-blank Question] *

Dependent on (Question: What types of alcohol do you usually drink? [Multiple Choice], first option)

Beer Drinking Frequency: ____ times/month, ____ bottles/time. [Fill-in-the-blank Question]*

Dependent on (Question: What types of alcohol did you usually drink before quitting? [Multiple Choice], first option)

Baijiu Drinking Frequency: ____ times/month, ____ liang/time. [Fill-in-the-blank Question] *

Dependent on (Question: What types of alcohol do you usually drink? [Multiple Choice], second option)

Baijiu Drinking Frequency: ____ times/month, ____ liang/time. [Fill-in-the-blank Question] *

Dependent on (Question: What types of alcohol did you usually drink before quitting? [Multiple Choice], second option)

Red Wine / Wine Drinking Frequency: ____ times/month, ____ liang/time. [Fill-in-the-blank Question] *

Dependent on (Question: What types of alcohol do you usually drink? [Multiple Choice], third option)

Red Wine / Wine Drinking Frequency: ____ times/month, ____ liang/time. [Fill-in-the-blank Question] *

Dependent on (Question: What types of alcohol did you usually drink before quitting? [Multiple Choice], third option)

Huangjiu / Rice Wine Drinking Frequency: ____ times/month, ____ liang/time. [Fill-in-the-blank Question] *

Dependent on (Question: What types of alcohol do you usually drink? [Multiple Choice], fourth option)

Other Alcohol Drinking Frequency: ____ times/month, ____ liang/time. [Fill-in-the-blank Question]*

Dependent on (Question: What types of alcohol did you usually drink before quitting? [Multiple Choice], fifth option)

Frequency of Getting Drunk from Alcohol: [Single Choice Question] *

○Almost every time ○Most of the time ○50% of the time ○Occasionally ○Never

Dependent on (Question: Do you drink alcohol [averaging at least 12 times per year over the past three years], first and second options)

Dietary Habits

When cooking at home, which type of cooking oil is mainly used? [Single Choice Question] *

○Soybean Oil ○Peanut Oil

○Rapeseed Oil ○Corn Oil

○Animal Fat ○Other __________________ *

○Unknown

When cooking at home, which type of fuel is mainly used? [Single Choice Question] *

○Wood Stove ○Coal Stove

○Induction Cooker ○Gas Stove

○Natural Gas ○Biogas

○Other __________________ *

When cooking at home, what is the main kitchen ventilation method? [Single Choice Question]*

○Range Hood ○Exhaust Fan

○Natural Window Ventilation ○Other __________________ *

When cooking at home, what is your preferred cooking method? [Single Choice Question] *

○Frying ○Boiling/Stewing

○Stir-frying ○Braising/Simmering

○Cold Dishes ○Smoking/Grilling

○Other __________________ *

Dietary Intake

Since entering university, on average per week (7 days), how many times do you eat breakfast, lunch, dinner, and late-night snacks? _______ times breakfast, _______ times lunch, _______ times dinner, _______ times late-night snack [Fill-in-the-blank Question]*

During your most recent holiday, on average per week, how many times do you eat breakfast, lunch, dinner, and late-night snacks? _______ times breakfast, _______ times lunch, _______ times dinner, _______ times late-night snack [Fill-in-the-blank Question] *

During high school, on average per week, how many times do you eat breakfast, lunch, dinner, and late-night snacks? _______ times breakfast, _______ times lunch, _______ times dinner, _______ times late-night snack [Fill-in-the-blank Question] *

During middle school, on average per week, how many times do you eat breakfast, lunch, dinner, and late-night snacks? _______ times breakfast, _______ times lunch, _______ times dinner, _______ times late-night snack [Fill-in-the-blank Question] *

During elementary school, on average per week, how many times do you eat breakfast, lunch, dinner, and late-night snacks? _______ times breakfast, _______ times lunch, _______ times dinner, _______ times late-night snack [Fill-in-the-blank Question] *

Your commonly consumed staple food is: [Single Choice Question] *

○Rice-based ○Wheat-based (Flour)

○Other _________________ *

Water Consumption

Your Daily Water Intake (1 cup = 250 ml) [Single Choice Question] *

○Less than 1 cup ○1–3 cups ○4–6 cups

○6~8 cups ○More than 8 cups

What type of water do you usually drink? [Multiple Choice Question] *

□Purified Water
□Bottled Water
□Mineral Water
□Tap Water
□Well Water
□River/Lake Water
□Other ________________*

Do you boil the water before drinking? [Single Choice Question] *

○Boiled
○Not boiled, drink directly

What temperature do you usually prefer your drinking water? [Single Choice Question] *

○Ice Wate
○Cool Water
○Room Temperature
○Warm
○Very Hot

What beverages do you often drink? [Multiple Choice Question] *

□Carbonated Drinks (Coke, Sprite, Fanta, etc.)
□Freshly Squeezed Juice
□Energy Drinks (Red Bull, etc.)
□Tea
□Coffee
□Milk Tea / Flavored Drinks from Shops
□Juice Drinks
□Rarely drink beverages

On average, how many times per week do you drink the following beverages? [Fill-in-the-blank Questions]*

Carbonated Drinks: ____ times/week

Dependent on (Question: What beverages do you often drink?, first option)

Freshly Squeezed Juice: ____ times/week [Fill-in-the-blank Questions] *

Dependent on (Question: What beverages do you often drink?, second option)

Energy Drinks: ____ times/week [Fill-in-the-blank Questions] *

Dependent on (Question: What beverages do you often drink?, third option)

Coffee: ____ times/week [Fill-in-the-blank Questions] *

Dependent on (Question: What beverages do you often drink?, fifth option)

Milk Tea / Flavored Drinks from Shops: ____ times/week [Fill-in-the-blank Questions] *

Dependent on (Question: What beverages do you often drink?, sixth option)

Juice Drinks: ____ times/week [Fill-in-the-blank Questions]*

Dependent on (Question: What beverages do you often drink?, seventh option)

Green Tea: ____ times/week (enter 0 if none) [Fill-in-the-blank Questions] *

Dependent on (Question: What beverages do you often drink?, fourth option)

Black Tea: ____ times/week (enter 0 if none) [Fill-in-the-blank Questions] *

Dependent on (Question: What beverages do you often drink?, fourth option)

Flower Tea: ____ times/week (enter 0 if none) [Fill-in-the-blank Questions] *

Dependent on (Question: What beverages do you often drink?, fourth option)

Other Tea (please specify type): _________ times/week (enter 0 if none) [Fill-in-the-blank Questions] *

Dependent on (Question: What beverages do you often drink?, fourth option.

Indoor Ventilation and Temperature Control

High School Classroom Cooling Equipment Installed: [Multiple Choice Question]*

□None □Air Conditioner □Electric Fan

Do high school classrooms use air conditioning in hot weather? [Single Choice Question] *

○Yes ○No

Dependent on (Question: High School Classroom Cooling Equipment Installed, second option)

What is the usual temperature setting of the air conditioner in hot weather? [Single Choice Question] *

○Below 20℃ ○20-25℃ ○25-28℃ ○Above 28℃

Dependent on (Question: Do high school classrooms use air conditioning in hot weather?, first option)

Middle School Classroom Cooling Equipment Installed: [Multiple Choice Question]*

□None □Air Conditioner □Electric Fan

Do middle school classrooms use air conditioning in hot weather? [Single Choice Question] *

○Yes ○No

Dependent on (Question: Middle School Classroom Cooling Equipment Installed, second option

What is the usual temperature setting of the air conditioner in hot weather? [Single Choice Question] *

○Below 20℃ ○20-25℃ ○25-28℃ ○Above 28℃

Dependent on (Question: Do middle school classrooms use air conditioning in hot weather?, first option)

Elementary School Classroom Cooling Equipment Installed: [Multiple Choice Question] *

□None □Air Conditioner □Electric Fan

Do elementary school classrooms use air conditioning in hot weather? [Single Choice Question] *

○Yes ○No

Dependent on (Question: Elementary School Classroom Cooling Equipment Installed, second option)

What is the usual temperature setting of the air conditioner in hot weather? [Single Choice Question] *

○Below 20℃ ○20-25℃ ○25-28℃ ○Above 28℃

Dependent on (Question: Do elementary school classrooms use air conditioning in hot weather?, first option)

Have you used air conditioning at home during hot weather in the past three years? [Single Choice Question]*

○Yes ○No

During summer, on average, how many _____ days per week do you use air conditioning at home, and how many hours _____ per day? [Fill-in-the-blank Question] *

Dependent on (Question: Have you used air conditioning at home during hot weather in the past three years?, first option)

What is the usual temperature setting of the air conditioner at home in hot weather? [Single Choice Question] *

○Below 20°C ○20-25°C ○25-28°C ○Above 28°C

Dependent on (Question: Have you used air conditioning at home during hot weather in the past three years?, first option)

Primary Heating Method in Middle School Classrooms: [Single Choice Question] *

○Central Heating ○Air Conditioner

○Coal Stove ○None

○Other, please specify _________________ *

Primary Heating Method in Elementary School Classrooms: [Single Choice Question] *

○Central Heating ○Air Conditioner

○Coal Stove ○None

○Other, please specify _________________ *

Primary Heating Method at Home in the Past Three Years: [Single Choice Question]*

○Central Heating ○Air Conditioner

○Coal Stove ○None

○Other, please specify _________________ *

Average Daily Outdoor Time in the Past Three Months: [Single Choice Question] *

○<1 hour ○1-3hours ○3-5hours ○>5hour

Have you exercised in the past three months (at least once per week)? [Single Choice Question] *

○Yes ○No

Sleep Condition

How would you rate the quality of your afternoon nap? [Single Choice Question] *

○No nap ○Very good ○Good ○Average ○Poor Very poor

Duration of Afternoon Nap: ____ minutes [Fill-in-the-blank Question *

Dependent on (Question: How would you rate the quality of your afternoon nap?, options 2–6)

Have you gone to bed after midnight in the past six months? [Single Choice Question] *

○Yes, average number of days per month: _________________ *

○No

In the past three months, you usually close your eyes to sleep at____: p.m., taking ___ minutes to fall asleep, and wake up at____: a.m. [Fill-in-the-blank Question] *

Do you usually look at your phone/tablet before sleep? [Single Choice Question] *

○Yes ○No

How long do you usually use your phone/tablet before sleep? [Single Choice Question] *

○ ≤30 minutes ○ 30–60 minutes ○> 60 minutes

Dependent on (Question: Do you usually look at your phone/tablet before sleep?, first option)*

Condition in the Past Month [Matrix Single Choice Question]

	Never	Occasionally (≤1 time/week)	Sometimes (1–2 times/week)	Often (≥3 times/week)
Difficulty falling asleep at night (unable to fall asleep within 30 minutes)	○	○	○	○
Easily awakened during sleep or early awakening (unable to fall back asleep after waking early)	○	○	○	○
Getting up at night to use the	○	○	○	○

toilet				
Shortness of breath during sleep	○	○	○	○
Coughing or snoring during sleep	○	○	○	○
Feeling cold during sleep	○	○	○	○
Feeling too hot during sleep	○	○	○	○
Having nightmares	○	○	○	○
Pain or physical discomfort during sleep	○	○	○	○
Other conditions affecting	○	○	○	○

sleep (if any, please specify):				
Need to take medication (prescribed or over-the-counter) to fall asleep	○	○	○	○
Difficulty staying awake while driving, eating, or engaging in social activities	○	○	○	○

Have you experienced any other conditions affecting your nighttime sleep? [Single Choice Question] *

○ None

○○ <1 time/week — please specify the reason: ___________________*

○1–2 times/week — please specify the reason: ___________________

○≥3 times/week — please specify the reason: ___________________ *

How would you rate your nighttime sleep quality? [Single Choice Question] *

○Very good ○Fairly good ○Average ○Poor ○Very poor

When doing things, are you able to maintain enthusiasm? [Single Choice Question] *

○Always ○ Usually ○Sometimes ○Rarely ○Never

Disease History, Medication History, and Family History

(Please fill in based on doctor or hospital diagnoses.)

Have you ever been diagnosed with the following diseases? [Multiple Choice Question] *

Ophthalmic Diseases

Ophthalmic Diseases

□ Conjunctivitis

□ Iritis

□ Myopia

□ Hyperopia

□ Other eye diseases: __________________ *

Respiratory System Diseases

□ Allergic rhinitis

□ Asthma

□ Chronic bronchitis

□ Pneumonia

□ Emphysema

□ Pulmonary tuberculosis

□ Other respiratory diseases: __________________ *

Digestive System Diseases

□ Gastrointestinal diseases

□ Liver diseases

□ Gallbladder diseases

□ Pancreatic diseases

□ Other digestive system diseases: __________________ *

Oral Diseases

□ Oral ulcers

□ Gingival bleeding

□ Dental caries (tooth decay)

□ Periodontitis

□ Other oral diseases: _________________ *

Urinary and Reproductive System Diseases

□ Nephritis

□ Kidney stones

□ Cystitis

□ Gout

□ Polycystic ovary syndrome (PCOS)

□ Other urinary/reproductive system diseases: _________________ *

Cardiovascular Diseases

□ Hypertension

□ Heart disease

□ Hyperlipidemia

□ Other cardiovascular diseases: _________________ *

Endocrine Diseases

□ Diabetes

□ Thyroid disease

□ Hashimoto’s thyroiditis

□ Other endocrine diseases: _________________ *

Mental Disorders

□ Depression

□ Anxiety

□ Mania

□ Schizophrenia

□ Obsessive-compulsive disorder (OCD)

□ Suicidal tendency

□ Other mental disorders: _________________ *

Dermatological Diseases

□ Eczema

□ Dermatitis

□ Vitiligo

□ Psoriasis

□ Systemic lupus erythematosus

□ Other skin diseases: _________________ *

Rheumatic and Autoimmune Diseases

□ Rheumatoid arthritis

□ Rheumatic heart disease

□ Other rheumatic/autoimmune diseases: _________________ *

Tumors

□ Leukemia

□ Lung cancer

□ Liver cancer

□ Gastric cancer

□ Bone cancer / Osteosarcoma

□ Other tumors: _________________ *

Neurological Diseases

□ Neuralgic headache

□ Dizziness

□ Epilepsy

□ Other neurological diseases: _________________ *

Other Medical Conditions

□ Other diseases: _________________ *

No Disease History

□ No disease history

Family History

Has your father ever been diagnosed with any of the following diseases? [Multiple Choice Question] *

Ophthalmic Diseases

□ Conjunctivitis

□ Iritis

□ Myopia

□ Hyperopia

□ Other eye diseases: _________________ *

Respiratory System Diseases

□ Allergic rhinitis

□ Asthma

□ Chronic bronchitis

□ Pneumonia

□ Emphysema

□ Pulmonary tuberculosis

□ Other respiratory diseases: _________________ *

Digestive System Diseases

□ Gastrointestinal diseases

□ Liver diseases

□ Gallbladder diseases

□ Pancreatic diseases

□ Other digestive system diseases: _________________ *

Oral Diseases

□ Oral ulcers

□ Gingival bleeding

□ Dental caries (tooth decay)

□ Periodontitis

□ Other oral diseases: _________________ *

Urinary and Reproductive System Diseases

□ Nephritis

□ Kidney stones

□ Cystitis

□ Gout

□ Polycystic ovary syndrome (PCOS)

□ Other urinary/reproductive system diseases: _________________ *

Cardiovascular Diseases

□ Hypertension

□ Heart disease

□ Hyperlipidemia

□ Other cardiovascular diseases: _________________ *

Endocrine Diseases

□ Diabetes

□ Thyroid disease

□ Hashimoto's thyroiditis

□ Other endocrine diseases: _________________ *

Mental Disorders

□ Depression

□ Anxiety

□ Mania

□ Schizophrenia

□ Obsessive-compulsive disorder (OCD)

□ Suicidal tendency

□ Other mental disorders: _________________ *

Dermatological Diseases

□ Eczema

□ Dermatitis

□ Vitiligo

□ Psoriasis

□ Systemic lupus erythematosus

□ Other skin diseases: _________________ *

Rheumatic and Autoimmune Diseases

□ Rheumatoid arthritis

□ Rheumatic heart disease

□ Other rheumatic/autoimmune diseases: _________________ *

Tumors

□ Leukemia

□ Lung cancer

□ Liver cancer

□ Gastric cancer

□ Bone cancer / Osteosarcoma

□ Other tumors: _________________ *

Neurological Diseases

□ Neuralgic headache

□ Dizziness

□ Epilepsy

□ Other neurological diseases: _________________ *

Other Medical Conditions

□ Other diseases: _________________ *

No Disease History

□ No disease history

Family History

Has your mother ever been diagnosed with the following diseases? [Multiple Choice Question] *

Ophthalmic Diseases

□ Conjunctivitis

□ Iritis

□ Myopia

□ Hyperopia

□ Other eye diseases: _________________ *

Respiratory System Diseases

□ Allergic rhinitis

□ Asthma

□ Chronic bronchitis

□ Pneumonia

□ Emphysema

□ Pulmonary tuberculosis

□ Other respiratory diseases: __________________ *

Digestive System Diseases

□ Gastrointestinal diseases

□ Liver diseases

□ Gallbladder diseases

□ Pancreatic diseases

□ Other digestive system diseases: __________________ *

Oral Diseases

□ Oral ulcers

□ Gingival bleeding

□ Dental caries (tooth decay)

□ Periodontitis

□ Other oral diseases: __________________ *

Urinary and Reproductive System Diseases

□ Nephritis

□ Kidney stones

□ Cystitis

□ Gout

□ Polycystic ovary syndrome (PCOS)

□ Other urinary/reproductive system diseases: __________________ *

Cardiovascular Diseases

□ Hypertension

□ Heart disease

□ Hyperlipidemia

□ Other cardiovascular diseases: __________________ *

Endocrine Diseases

□ Diabetes

□ Thyroid disease

□ Hashimoto’s thyroiditis

□ Other endocrine diseases: _________________ *

Mental Disorders

□ Depression

□ Anxiety

□ Mania

□ Schizophrenia

□ Obsessive-compulsive disorder (OCD)

□ Suicidal tendency

□ Other mental disorders: _________________ *

Dermatological Diseases

□ Eczema

□ Dermatitis

□ Vitiligo

□ Psoriasis

□ Systemic lupus erythematosus

□ Other skin diseases: _________________ *

Rheumatic and Autoimmune Diseases

□ Rheumatoid arthritis

□ Rheumatic heart disease

□ Other rheumatic/autoimmune diseases: _________________ *

Tumors

□ Leukemia

□ Lung cancer

□ Liver cancer

□ Gastric cancer

□ Bone cancer / Osteosarcoma

□ Other tumors: _________________ *

Neurological Diseases

□ Neuralgic headache

□ Dizziness

□ Epilepsy

□ Other neurological diseases: ________________ *

Other Medical Conditions

□ Other diseases: ________________ *

No Disease History

□ No disease history

Have the following diseases been treated？

If treated, please specify the medication or method used. If you do not remember, please write “0.”[Matrix Single Choice Question*

	Untreated	Medication Treatment	Surgical Treatment
Appearance	○	○	○
Function	○	○	○

This question is displayed only if the respondent selected any option (1–63) in “Have you ever been diagnosed with any of the following diseases? (Multiple Choice Question)”

Hospitalization in the Past Three Years (add rows if needed): [Table Text Question]

	Hospitalization Date	Reason for Hospitalization
1		
2		
3		
4		
5		
6		
7		
8		

9		
10		

Have you experienced any illness or injury in the past two weeks? [Single Choice Question] *

○No ○Yes, reason: _________________ *

Medical Treatment: [Single Choice Question] *

○Did not seek medical attention, nor took any medication or alternative therapy

○Did not seek medical attention, nor took any medication or alternative therapy

○Sought medical treatment at a healthcare facility

Depends on (Question: "Have you experienced any illness or injury in the past two weeks?") Option 2

Age at First Menstruation: [Single Choice Question]* *

○(years) _________________ * ○None

Depends on (Question: "Gender") Option 1

Menstrual cycle (the interval between two consecutive periods): _____ days to _____ days; each period lasts about _____ days; menstrual flow is _____. [Fill-in-the-blank Question] *

Depends on (Question: "Gender") Option 1

Do you experience dysmenorrhea (menstrual pain)? [Single Choice Question]* *

○No ○Yes

Depends on (Question: “Age at First Menstruation”) Option 1

Degree of Dysmenorrhea: [Single Choice Question] *

○Mild ○Moderate ○Severe

Depends on (Question: “Do you experience dysmenorrhea (menstrual pain)?”) Option 2

Emotion and Stress

In the past two weeks, how much psychological stress have you felt? [Single Choice Question] *

○None ○A little ○Moderate ○Quite a lot ○Very high

Please indicate your condition in the past two weeks: [Matrix Single Choice Question] *

	Not at all	Several days	More than half the days	Nearly every day
Little interest or pleasure in doing things	○	○	○	○
Feeling down, depressed, or hopeless	○	○	○	○
Trouble falling or staying asleep, or sleeping too	○	○	○	○

much				
Feeling tired or having little energy	○	○	○	○
Poor appetite or overeating	○	○	○	○
Feeling bad about yourself — or that you are a failure or have let yourself or your family down	○	○	○	○
Trouble concentrating on things, such as reading the newspaper or watching television	○	○	○	○
Moving or speaking so slowly that	○	○	○	○

other people could have noticed, or the opposite — being so fidgety or restless that you have been moving around a lot more than usual				
Thoughts that you would be better off dead or of hurting yourself in some way	○	○	○	○

Please indicate your condition in the past two weeks: [Matrix Single Choice Question] *

	Not at all	Several days	More than half the days	Nearly every day
Feeling nervous,	○	○	○	○

anxious, or on edge				
Not being able to stop or control worrying	○	○	○	○
Not being able to stop or control worrying	○	○	○	○
Trouble relaxing	○	○	○	○
Being so restless that it’s hard to sit still	○	○	○	○
Becoming easily annoyed or irritable	○	○	○	○
Feeling afraid as if something awful might	○	○	○	○

happen				

滨州医学院大学生健康影响因素研究（第一次）

知情同意书

亲爱的同学，您好：

本问卷主要进行环境暴露与健康影响的流行病学研究。由于各位同学入学前来自不同地区，环境污染物暴露情况不同，体内污染物含量不同，对健康的影响也不一样。基于这一特点，本课题组诚挚邀请大家参与本次"环境污染物体内暴露情况调查研究"，探究不同污染物可能对我们的身体造成怎样的伤害。调查结果将提交给本研究组作进一步研究分析，个人信息不会以任何形式泄露给第三方。您可以自由选择是否参与此项研究，也可以随时退出。如果您中途退出此项研究，项目组将会中止对您收集信息。 [单选题] *

○我已经阅读了该知情同意书，愿意参与本研究

姓名 [填空题] *

____

性别 [单选题] *

○女　　○男

基本信息：[矩阵文本题] *

民族：	________________________
出生日期：	________________________
身份证	________________________

号：	
学号：	________________________
联系电话：	________________________
QQ 号：	________________________
微信号：	________________________
身高(cm)：	________________________
体重(kg)：	________________________
您平均一天看多长时间手机/平板（分钟）	________________________

入学前信息

大学入学前家庭住址：________________________街道（乡、村）：________________ [填空题] *

高中学校名称（以在校时间最长学校为准）：________________

地址：________________________街道（乡、村）：________________

是否住校：___ [填空题] *

初中学校名称（以在校时间最长学校为准）：________________

地址：__________________________街道（乡、村）：________________

是否住校：___ [填空题] *

小学学校名称（以在校时间最长学校为准）：________________

地址：__________________________街道（乡、村）：________________

是否住校：___ [填空题] *

出生时家庭地址：__________________________街道（乡、村）：________________

现家庭人口数：___

去年您全家总收入为：________ [填空题] *

吸烟情况

您是否吸烟（吸烟指平均每天至少吸一支并持续半年以上）：[单选题] *

○吸烟　　○已戒烟

○从不吸烟或偶尔吸烟（平均每天不超过一支）

吸烟情况[矩阵文本题] *

开始吸烟年龄：	__________________________
现在平均	__________________________

每天吸烟数（支）：	

依赖于（题目：您是否吸烟（吸烟指平均每天至少吸一支并持...）第 1 个选项

吸烟情况[矩阵文本题] *

开始吸烟年龄：	________________
戒烟年龄：	________________
戒烟前平均每天吸烟数（支）：	________________

依赖于（题目：您是否吸烟（吸烟指平均每天至少吸一支并持...）第 2 个选项

您有没有经常吸入别人吸烟产生的烟雾（平均每天吸入其他吸烟者呼出烟雾 15 分钟以上）？ [单选题] *

○没有或基本没有 ○有

依赖于（题目：您是否吸烟（吸烟指平均每天至少吸一支并持...）第 3 个选项

您平均每天被动吸烟时长约___小时___分钟。

您被动吸烟了___年。 [填空题] *

依赖于（题目：您有没有经常吸入别人吸烟产生的烟雾（平均...）第 2 个选项

饮酒情况

您是否饮酒（近三年平均每年至少饮酒 12 次以上）：[单选题] *

○饮酒　　○已戒酒

○不饮酒或偶尔饮酒

饮酒情况[矩阵文本题] *

开始饮酒年龄：	________________
戒酒年龄：	________________

依赖于（题目：您是否饮酒（近三年平均每年至少饮酒 12 次...）第 2 个选项

您经常喝什么类型的酒？（多选）[多选题] *

□啤酒　　□白酒

□红酒/葡萄酒　　□黄酒/米酒

□其它 ________________*

依赖于（题目：您是否饮酒（近三年平均每年至少饮酒 12 次...）第 1 个选项

您戒酒前经常喝什么类型的酒？（多选）[多选题] *

□啤酒　　□白酒

□红酒/葡萄酒　　□黄酒/米酒

□其它 ________________*

依赖于（题目：您是否饮酒（近三年平均每年至少饮酒 12 次...）第 2 个选项

啤酒饮用频率：____次/月，____瓶/次。 [填空题] *

依赖于（题目：您经常喝什么类型的酒？（多选））第 1 个选项

啤酒饮用频率：____次/月，____瓶/次。 [填空题] *

依赖于（题目：您戒酒前经常喝什么类型的酒？（多选））第 1 个选项

白酒饮用频率：____次/月，____两/次。 [填空题] *

依赖于（题目：您经常喝什么类型的酒？（多选））第 2 个选项

白酒饮用频率：____次/月，____两/次。 [填空题] *

依赖于（题目：您戒酒前经常喝什么类型的酒？（多选））第 2 个选项

红酒/葡萄酒饮用频率：____次/月，____两/次。 [填空题] *

依赖于（题目：您经常喝什么类型的酒？（多选））第 3 个选项

红酒/葡萄酒饮用频率：____次/月，____两/次。 [填空题] *

依赖于（题目：您戒酒前经常喝什么类型的酒？（多选））第 3 个选项

黄酒/米酒饮用频率：____次/月，____两/次。 [填空题] *

依赖于（题目：您经常喝什么类型的酒？（多选））第 4 个选项

黄酒/米酒饮用频率：____次/月，____两/次。 [填空题] *

依赖于（题目：您戒酒前经常喝什么类型的酒？（多选））第 4 个选项

其它酒饮用频率：____次/月，____两/次。 [填空题] *

依赖于（题目：您戒酒前经常喝什么类型的酒？（多选））第 5 个选项

您饮酒喝醉的频率 [单选题] *

○几乎每次 ○绝大多数 ○50%几率 ○偶尔 ○从不

依赖于（题目：您是否饮酒（近三年平均每年至少饮酒 12 次...）第 1;2 个选项

膳食习惯

您家里做饭时，主要使用哪种食用油(单选)？ [单选题] *

○豆油 ○花生油

○菜籽油 ○玉米油

○动物油 ○其它 _________________ *

○不详

您家里做饭时，主要使用哪种燃料(单选)？ [单选题] *

○柴灶 ○煤炉灶

○电磁炉 ○煤气

○天然气 ○沼气

○其它 _________________ *

您家里做饭时，厨房主要的通风方式(单选) [单选题] *

○抽油烟机 ○排风扇

○自然开窗通风 ○其它 ________________ *

您家里做饭时，最喜欢的烹调方式(单选) [单选题] *

○煎炸 ○烧煮

○清炒 ○炖煨

○凉拌 ○熏烤

○其它 ________________ *

膳食情况

您大学入学以来，平均一周（7 天）吃_______次早饭，_______次午饭，_______次晚饭，______次夜宵。 [填空题] *

您在最近的假期中，平均一周吃_______次早饭，_______次午饭，_______次晚饭，______次夜宵。 [填空题] *

您在高中时期，平均一周吃_______次早饭，_______次中饭，_______次晚饭，______次夜宵。 [填空题] *

您在初中时期，平均一周吃_______次早饭，_______次午饭，_______次晚饭，______次夜宵。 [填空题] *

您在小学时期，平均一周吃________次早饭，________次午饭，________次晚饭，_______次夜宵。 [填空题] *

您常食用的主食是： [单选题] *

○大米类
○面食类（小麦粉）
○其它 ____________________ *

饮水情况

您每天饮水量(250ml 一杯) [单选题] *

○1 杯以下
○1~3 杯
○4~6 杯
○6~8 杯
○8 杯以上

您平时主要喝哪种水？ [多选题] *

□净化过的水
□桶装水
□矿泉水
□自来水
□井水
□河湖水
□其他 ____________________*

是否烧开后再喝？ [单选题] *

○烧开水
○不烧开，直接饮用

您饮水时习惯什么温度？ [单选题] *

○冰水
○凉水
○常温
○偏热
○很热

您经常喝什么饮料？ [多选题] *

□碳酸饮料(可乐、雪碧、芬达等)
□鲜榨果汁
□功能饮料(红牛等)
□茶
□咖啡
□奶茶店勾兑风味饮料
□果汁饮料
□几乎不喝饮料

您平均每周喝____次碳酸饮料。 [填空题] *

依赖于（题目：您经常喝什么饮料？）第 1 个选项

您平均每周喝____次鲜榨果汁。 [填空题] *

依赖于（题目：您经常喝什么饮料？）第 2 个选项

您平均每周喝____次功能饮料。 [填空题] *

依赖于（题目：您经常喝什么饮料？）第 3 个选项

您平均每周喝____次咖啡。 [填空题] *

依赖于（题目：您经常喝什么饮料？）第 5 个选项

您平均每周喝____次奶茶店勾兑风味饮料。 [填空题] *

依赖于（题目：您经常喝什么饮料？）第 6 个选项

您平均每周喝____次果汁饮料。 [填空题] *

依赖于（题目：您经常喝什么饮料？）第 7 个选项

您平均每周喝____次绿茶（无请填 0）。 [填空题] *

依赖于（题目：您经常喝什么饮料？）第 4 个选项

您平均每周喝____次红茶（无请填 0）。 [填空题] *

依赖于（题目：您经常喝什么饮料？）第 4 个选项

您平均每周喝____次花茶（无请填 0）。 [填空题] *

依赖于（题目：您经常喝什么饮料？）第 4 个选项

您平均每周喝____次其它茶，请注明种类：_________（无请填 0）。 [填空题] *

依赖于（题目：您经常喝什么饮料？）第 4 个选项

室内通风情况

高中教室防暑降温设备安装情况： [多选题] *

□没装　　□空调　　□电风扇

高中教室热天是否使用空调？ [单选题] *

○是　　○否

依赖于（题目：高中教室防暑降温设备安装情况：）第 2 个选项

高中教室热天使用空调时常设的温度是多少？ [单选题] *

○20℃以下　　○20-25℃　　○25-28℃　　○28℃以上

依赖于（题目：高中教室热天是否使用空调？）第 1 个选项

初中教室防暑降温设备安装情况：[多选题] *

□没装 □空调 □电风扇

初中教室热天是否使用空调？[单选题] *

○是 ○否

依赖于（题目：初中教室防暑降温设备安装情况：）第 2 个选项

初中教室热天使用空调时常设的温度是多少？[单选题] *

○20℃以下 ○20-25℃ ○25-28℃ ○28℃以上

依赖于（题目：初中教室热天是否使用空调？）第 1 个选项

小学教室防暑降温设备安装情况：[多选题] *

□没装 □空调 □电风扇

小学教室热天是否使用空调？[单选题] *

○是 ○否

依赖于（题目：小学教室防暑降温设备安装情况：）第 2 个选项

小学教室热天使用空调时常设的温度是多少？[单选题] *

○20℃以下 ○20-25℃ ○25-28℃ ○28℃以上

依赖于（题目：小学教室热天是否使用空调？）第 1 个选项

您家中近三年热天是否使用空调？[单选题] *

○是 ○否

您家中夏季平均开___天空调，每天平均开___小时。[填空题] *

依赖于（题目：您家中近三年热天是否使用空调？）第 1 个选项

家中热天使用空调时常设的温度是多少？ [单选题] *

○20℃以下 ○20-25℃ ○25-28℃ ○28℃以上

依赖于（题目：您家中近三年热天是否使用空调？）第 1 个选项

高中教室主要使用哪种方式取暖？ [单选题] *

○暖气 ○空调

○煤炉 ○没有

○其他,请注明 _________________ *

初中教室使用哪种方式取暖？ [单选题] *

○暖气 ○空调

○煤炉 ○没有

○其他,请注明 _________________ *

小学教室使用哪种方式取暖？ [单选题] *

○暖气 ○空调

○煤炉 ○没有

○其他,请注明 _________________ *

家中近三年使用哪种方式取暖？ [单选题] *

○暖气 ○空调

○煤炉 ○没有

○其他,请注明 _________________ *

您最近三个月的平均每天户外停留时间是： [单选题] *

○<1 小时　○1-3 小时　○3-5 小时　○>5 小时

近三个月是否锻炼身体(至少每周锻炼一次) [单选题] *

○是　○否

睡眠情况

您感觉您的午休睡眠质量怎么样？ [单选题] *

○不午休　○很好　○较好　○一般　○较差　○很差

午休睡眠时长:____分钟 [填空题] *

依赖于（题目：您感觉您的午休睡眠质量怎么样？）第 2;3;4;5;6 个选项

您最近半年是否有过晚上 12 点之后入睡？ [单选题] *

○是，平均每月天数： _________________ *

○否

您最近三个月通常晚上___点___分闭眼准备睡觉，需要___ 分钟才能入睡。早上___点___分醒来。 [填空题] *

您晚上睡前是否习惯看手机/平板？ [单选题] *

○是　　○否

您睡前一般看多长时间手机/平板？ [单选题] *

○≤30 分钟　　○30-60 分钟　　○>60 分钟

依赖于（题目：您晚上睡前是否习惯看手机/平板？）第 1 个选项

您最近一个月的睡眠情况[矩阵单选题] *

	无	<1 次/周	1-2 次/周	≥3 次/周
夜间入睡困难(指30min 内不能入睡)	○	○	○	○
夜间睡眠过程中易醒或早醒（凌晨醒后不容易再次入睡）	○	○	○	○
夜间起床去厕所	○	○	○	○
夜间睡眠时呼吸不畅	○	○	○	○

夜间睡眠时咳嗽或打鼾	○	○	○	○
夜间睡眠时感觉寒冷	○	○	○	○
夜间睡眠时感觉太热	○	○	○	○
夜间睡眠时做噩梦	○	○	○	○
夜间睡眠时疼痛不适	○	○	○	○
其它影响夜间睡眠的情况（如有，请说明？）	○	○	○	○
需要服药（包括从医生处方或者在外面药店购	○	○	○	○

买）才能入睡？				
在开车、吃饭或参加社会活动时难以保持清醒状态	○	○	○	○

是否有其它影响夜间睡眠的情况？ [单选题] *

○无

○<1 次/周，请注明原因：________ *

○1-2 次/周，请注明原因：________ *

○≥3 次/周，请注明原因：________ *

您感觉您的夜间睡眠质量怎么样？ [单选题] *

○很好 ○较好 ○一般 ○较差 ○很差

您做事情时，是否能保持热情？ [单选题] *

○可以 ○还可以 ○勉强可以 ○几乎不能 ○不能

疾病史、用药史与家族史（请根据医生/医院确诊结果填写）

您是否患过以下疾病？可多选。 [多选题] *

眼科疾病

□结膜炎

□虹膜炎

□近视

□远视

□其他眼科疾病: ___________________*

呼吸系统疾病

□过敏性鼻炎

□哮喘

□慢性支气管炎

□肺炎

□肺气肿

□肺结核

□其他呼吸系统疾病: ___________________*

消化系统疾病

□胃肠疾病

□肝脏疾病

□胆囊疾病

□胰腺疾病

□其他消化系统疾病: ___________________*

口腔疾病

□口腔溃疡

□牙龈出血

□龋齿

□牙周炎

□其他口腔疾病: ___________________*

泌尿生殖系统疾病

□肾炎

□肾结石

□膀胱炎

□痛风

□多囊卵巢

□其他泌尿生殖系统疾病: ________________*

心血管系统疾病

□高血压

□心脏病

□高脂血症

□其他心血管系统疾病: ________________*

内分泌系统疾病

□糖尿病

□甲状腺疾病

□桥本氏甲状腺炎(？)

□其他内分泌系统疾病: ________________*

精神疾病

□抑郁

□焦虑

□躁狂

□精神分裂

□强迫症

□自杀倾向

□其他精神疾病: ________________*

皮肤病

□湿疹

□皮炎

□白癜风

□银屑病(牛皮癣)

□系统性红斑狼疮

□其他皮肤病: ________________*

风湿免疫性疾病

□风湿性关节炎

□风湿性心脏病

□其他风湿免疫性疾病: ________________*

肿瘤

□白血病

□肺癌

□肝癌

□胃癌

□骨癌/骨肉瘤

□其他肿瘤: ________________*

神经系统疾病

□神经性头痛

□头晕

□癫痫

□其它神经系统疾病 ________________*

其它疾病史

□其它疾病史 ________________*

无疾病史

□无疾病史

家族史：

您父亲是否患过以下疾病？可多选。 [多选题] *

眼科疾病

□结膜炎

□虹膜炎

□近视

□远视

□其他眼科疾病: ____________________*

呼吸系统疾病

□过敏性鼻炎

□哮喘

□慢性支气管炎

□肺炎

□肺气肿

□肺结核

□其他呼吸系统疾病: ____________________*

消化系统疾病

□胃肠疾病

□肝脏疾病

□胆囊疾病

□胰腺疾病

□其他消化系统疾病: ________________*

口腔疾病

□口腔溃疡

□牙龈出血

□龋齿

□牙周炎

□其他口腔疾病: ________________*

泌尿生殖系统疾病

□肾炎

□肾结石

□膀胱炎

□痛风

□多囊卵巢

□其他泌尿生殖系统疾病: ________________*

心血管系统疾病

□高血压

□心脏病

□高脂血症

□其他心血管系统疾病: ________________*

内分泌系统疾病

□糖尿病

□甲状腺疾病

□桥本氏甲状腺炎(？)

□其他内分泌系统疾病: ________________*

精神疾病

□抑郁

□焦虑

□躁狂

□精神分裂

□强迫症

□自杀倾向

□其他精神疾病: ________________*

皮肤病

□湿疹

□皮炎

□白癜风

□银屑病(牛皮癣)

□系统性红斑狼疮

□其他皮肤病: ________________*

风湿免疫性疾病

□风湿性关节炎

□风湿性心脏病

□其他风湿免疫性疾病: ________________*

肿瘤

□白血病

□肺癌

□肝癌

□胃癌

□骨癌/骨肉瘤

□其他肿瘤: ________________*

神经系统疾病

□神经性头痛

□头晕

□癫痫

□其它神经系统疾病 ________________*

其它疾病史

□其它疾病史 ________________*

无疾病史

□无疾病史

家族史：

您母亲是否患过以下疾病？可多选。 [多选题] *

眼科疾病

□结膜炎

□虹膜炎

□近视

□远视

□其他眼科疾病: ________________*

呼吸系统疾病

□过敏性鼻炎

□哮喘

□慢性支气管炎

□肺炎

□肺气肿

□肺结核

□其他呼吸系统疾病: ____________________*

消化系统疾病

□胃肠疾病

□肝脏疾病

□胆囊疾病

□胰腺疾病

□其他消化系统疾病: ____________________*

口腔疾病

□口腔溃疡

□牙龈出血

□龋齿

□牙周炎

□其他口腔疾病: ____________________*

泌尿生殖系统疾病

□肾炎

□肾结石

□膀胱炎

□痛风

□多囊卵巢

□其他泌尿生殖系统疾病: ____________________*

心血管系统疾病

□高血压

□心脏病

□高脂血症

□其他心血管系统疾病: __________________*

内分泌系统疾病

□糖尿病

□甲状腺疾病

□桥本氏甲状腺炎(？)

□其他内分泌系统疾病: __________________*

精神疾病

□抑郁

□焦虑

□躁狂

□精神分裂

□强迫症

□自杀倾向

□其他精神疾病: __________________*

皮肤病

□湿疹

□皮炎

□白癜风

□银屑病(牛皮癣)

□系统性红斑狼疮

□其他皮肤病: __________________*

风湿免疫性疾病

□风湿性关节炎

□风湿性心脏病

□其他风湿免疫性疾病: ___________________*

肿瘤

□白血病

□肺癌

□肝癌

□胃癌

□骨癌/骨肉瘤

□其他肿瘤: ___________________*

神经系统疾病

□神经性头痛

□头晕

□癫痫

□其它神经系统疾病 ___________________*

其它疾病史

□其它疾病史 ___________________*

无疾病史

□无疾病史

以下疾病是否经过治疗？

经过治疗请注明治疗药物或方法，记不清请填 0。[矩阵单选题] *

	未治疗	药物治疗	手术治疗
外观	○	○	○
功能	○	○	○

依赖于（题目：您是否患过以下疾病？可多选。）第 1;2;3;4;5;6;7;8;9;10;11;12;13;14;15;16;17;18;19;20;21;22;23;24;25;26;27;28;29;30;31;32;33;34;35;36;37;38;39;40;41;42;43;44;45;46;47;48;49;50;51;52;53;54;55;56;57;58;59;60;61;62;63 个选项

近三年住院情况（可加行）：[表格文本题]

	住院日期	住院原因
1		
2		
3		
4		
5		
6		
7		
8		
9		
10		

最近两周，您的身体是否有病伤的情况？ [单选题] *

○否 ○是，原因：__________________ *

诊疗情况： [单选题] *

○没有就诊，也没有自服药物或采取辅助疗法

○没有就诊，但自服了药物或采取了一些辅助疗法

○去医疗卫生单位诊疗

依赖于（题目：最近两周，您的身体是否有病伤的情况？）第 2 个选项

初次月经年龄：[单选题] *

○（岁）__________________ *　　　○无

依赖于（题目：性别）第 1 个选项

月经周期（相邻月经的间隔天数）约:_____天～______天，每次月经持续时间约:_____天，月经量______。[填空题] *

依赖于（题目：性别）第 1 个选项

是否有痛经：[单选题] *

○否　　　○是

依赖于（题目：初次月经年龄：）第 1 个选项

痛经程度：[单选题] *

○轻微　　　○一般　　　○很痛

依赖于（题目：是否有痛经：）第 2 个选项

情绪与压力

最近 2 周，您感觉您的心理压力有多大？[单选题] *

○没有　　　○有一点　　　○一般　　　○比较大　　　○很大

请填写您最近 2 周的情况：[矩阵单选题] *

	完全不会	好几天	一半以上	几乎每天
做事时提不起劲或没有兴趣	○	○	○	○
感到情绪低落、沮丧或绝望	○	○	○	○
入睡困难、睡不安稳或睡眠过多	○	○	○	○
感觉疲倦或没有活力	○	○	○	○
食欲不振或吃太多	○	○	○	○
觉得自己很糟或觉得自己很失败，或让自己或家人失望	○	○	○	○
对事物专注有困难，例如	○	○	○	○

阅读报纸或看电视时				
动作或说话速度缓慢到别人已经察觉，或正好相反——烦躁或坐立不安、来回走动的情况更胜于平常	○	○	○	○
有“不如死掉”或用某种方式伤害自己的念头	○	○	○	○

请填写您最近 2 周的情况：[矩阵单选题] *

	从来没有	有几天	刚超过一般天数	接近每天
感觉心神不安、焦虑或高度	○	○	○	○

紧张				
不能停止或控制担心	○	○	○	○
为各种各样的事情过度担心	○	○	○	○
难以放松	○	○	○	○
非常不安静以至于难以坐定	○	○	○	○
变得易恼火或易急躁	○	○	○	○
害怕发生可怕的事情	○	○	○	○
