# Supplementary material for: Metabolic characteristics related to potentially toxic elements in the blood of young adults in China: a cross-sectional study
Source: Front Nutr. 2025 Nov 19;12:1678706. doi: 10.3389/fnut.2025.1678706 (PMC12672240; doi:10.3389/fnut.2025.1678706)
Supplement: Supplementary file 6 [file Table_5.DOCX]

**Supplementary material**

**Metabolic characteristics related to potentially toxic elements in the blood of young adults in China: A cross-sectional study**

Lei Zhang^a^; Wen Xu^b^; Mingyu Feng^a^; Jia Zhang^a^; Zhenzhong Wang^a^; Wenfeng Kang^a^; Yi Liu^a^; Beibei Yang^c^; Yuming Guo^b^; Peng Lu^a^*

^a^ School of Public Health, Binzhou Medical University, Yantai, Shandong, China

^b^ Department of Epidemiology and Preventive Medicine, School of Public Health and Preventive Medicine, Monash University, Melbourne, Victoria, Australia

^c^ Yantai Affiliated Hospital of Binzhou Medical University, Yantai, Shandong, China

^*^ Corresponding author: Prof. P. Lu

Address: School of Public Health, Binzhou Medical University, No. 346 Guanhai Road, Laishan District, Yantai 264003, Shandong, China

Tel: +86–0535–6913272

Email: peng.lu@monash.edu

***Measurement of blood metal concentrations***

Specifically, 0.35 mL of whole blood samples were transferred to a quartz tube, followed by the addition of 0.40 mL of nitric acid. The quartz tubes were predigested at room temperature for two hours and then placed in an Ultra WAVE microwave digestion system (Ultra WAVE, Milestone Co, Italy) under programmed temperature conditions for 50 min. Subsequently, 0.10 mL of internal standard indium (2 ng/mL, In and Re) was added to the sample, which was then diluted to a final volume of 8 mL with ultrapure water. Then, an Elan DRC II ICP-MS instrument (PerkinElmer Sciex, USA) was utilized to measured blood metal concentrations. Helium gas fow rate: 4.5 mL/min, atomizing gas (Argon) flow rate: 1.05 L/min, auxiliary gas flow rate: 1.80L /min, plasma gas flow rate: 18.0 L /min, RF generator power: 1100w, residence time: 50-100ms, sample lifting volume: 1.1 mL/min, scanning mode: single-point peak hopping, resolution: 0.7-0.9 amu.

Quality assurance and quality control (QA/QC) were conducted in this study. Reagent blanks and procedural blanks were prepared along with each analysis batch to eliminate potential background contamination. The limit of detection (LOD) for metal was calculated as three times the standard deviation (SD) of the replicate measurements (10 times) of the procedural blank samples. The concentrations of metals below the limit of detection (LOD) are substituted with LOD/$\sqrt{2}$.

***Metabolomics and Lipidomics Measurements***

Metabolomics and lipidomics analyses were conducted using an Ultimate 3000 UHPLC system coupled with a Q-Exactive HF mass spectrometer. For the metabolomics component, the aqueous phase was separated on an Xbridge Amide column (100 × 2.1 mm i.d., 3.5 μm; Waters, USA) maintained at 30°C. The mobile phase consisted of 5% acetonitrile in water (solvent A) and 5% water in acetonitrile (solvent B), both supplemented with 10 mM ammonium acetate and 0.1% ammonia. The separation was executed at a flow rate of 0.35 mL/min using a linear gradient: 95% B from 0 to 0.5 min, 50% B at 14 min, maintaining 50% B until 15.5 min, followed by a return to 95% B at 16 min, and holding until 19 min. Samples were prepared by suspending them in 100 μL of a 1:1 (v/v) acetonitrile/water solution, with 10 μL of this solution injected for analysis.

For lipid analysis, chromatographic separation was achieved using a reversed-phase X-select CSH C18 column (2.1 mm × 100 mm, 2.5 μm; Waters, USA) at 40°C. The gradient elution was performed with two solvents containing 10 mM ammonium formate and 0.1% formic acid: ACN/water (3:2, v/v) and IPA/ACN (9:1, v/v). The gradient protocol included: 40% B at 0 min, 43% B at 2 min, 60% B at 12 min, 75% B at 12.1 min, 99% B at 18 min, and returning to 40% B at 20 min, with a total runtime of 23 min. The flow rate was set at 0.4 mL/min. Samples were reconstituted in a chloroform/methanol solution (1:1, v/v) and subsequently diluted threefold with an isopropanol/acetonitrile/H_2_O solution (2:1:1, v/v), with a 10 μL injection volume.

Mass spectrometric analysis was performed on the Q-Exactive HF (Thermo Scientific) using data-dependent acquisition (DDA) in positive-negative ion switching mode. Each cycle involved one survey scan (MS1) at a resolution of 60,000 over the range of 60 to 900 m/z for hydrophilic metabolites and 240 to 1200 m/z for lipids, followed by 10 MS/MS scans in HCD mode at a resolution of 15,000, utilizing stepped normalized collision energies of 15, 30, and 45. Dynamic exclusion was set to 10 seconds. For MS1 and MS/MS scans, the automatic gain control (AGC) targets were set at 5e6 (maximum injection time of 30 ms) and 2e5 (maximum injection time of 80 ms), respectively. Ion source settings included a spray voltage of 3.3 kV for positive ion mode and 3.0 kV for negative ion mode, with sheath gas at 40, auxiliary gas at 10, a capillary temperature of 320 °C, a probe heater temperature of 300 °C, and a Slens RF level of 55. A total of 2019 blood samples (n=581) were analyzed in a random sequence.

***Metabolites and metabolic pathways associated with Fe***

A total of 18 metabolites were enriched in 7 significant metabolic pathways. As illustrated in **Fig. S6**, elevated Fe levels were associated with upregulation of the Tyrosine, 3-Methyl-2-oxobutanoic acid, 4-Methyl-2-oxopentanoate, Quinolinate, 4-Pyridoxate, Arginine, Citrulline, Glutamate, Ornithine, N-Acetylornithine, Histamine, Histidine. Conversely, 10 metabolites exhibited downregulated expression in association with high blood Fe levels: Nicotinamide, Nicotinate, Glutamine, Pyridoxal, Pyridoxine. These pathways included Arginine biosynthesis, Phenylalanine metabolism, Histidine metabolism, Nicotinate and nicotinamide metabolism, Valine, leucine and isoleucine biosynthesis, Phenylalanine, tyrosine and tryptophan biosynthesis, Pyrimidine metabolism, Nitrogen metabolism, Glutathione metabolism, Glyoxylate and dicarboxylate metabolism, Glycine, serine and threonine metabolism, Pantothenate and CoA biosynthesis and Vitamin B6 metabolism (**Fig. S7**).


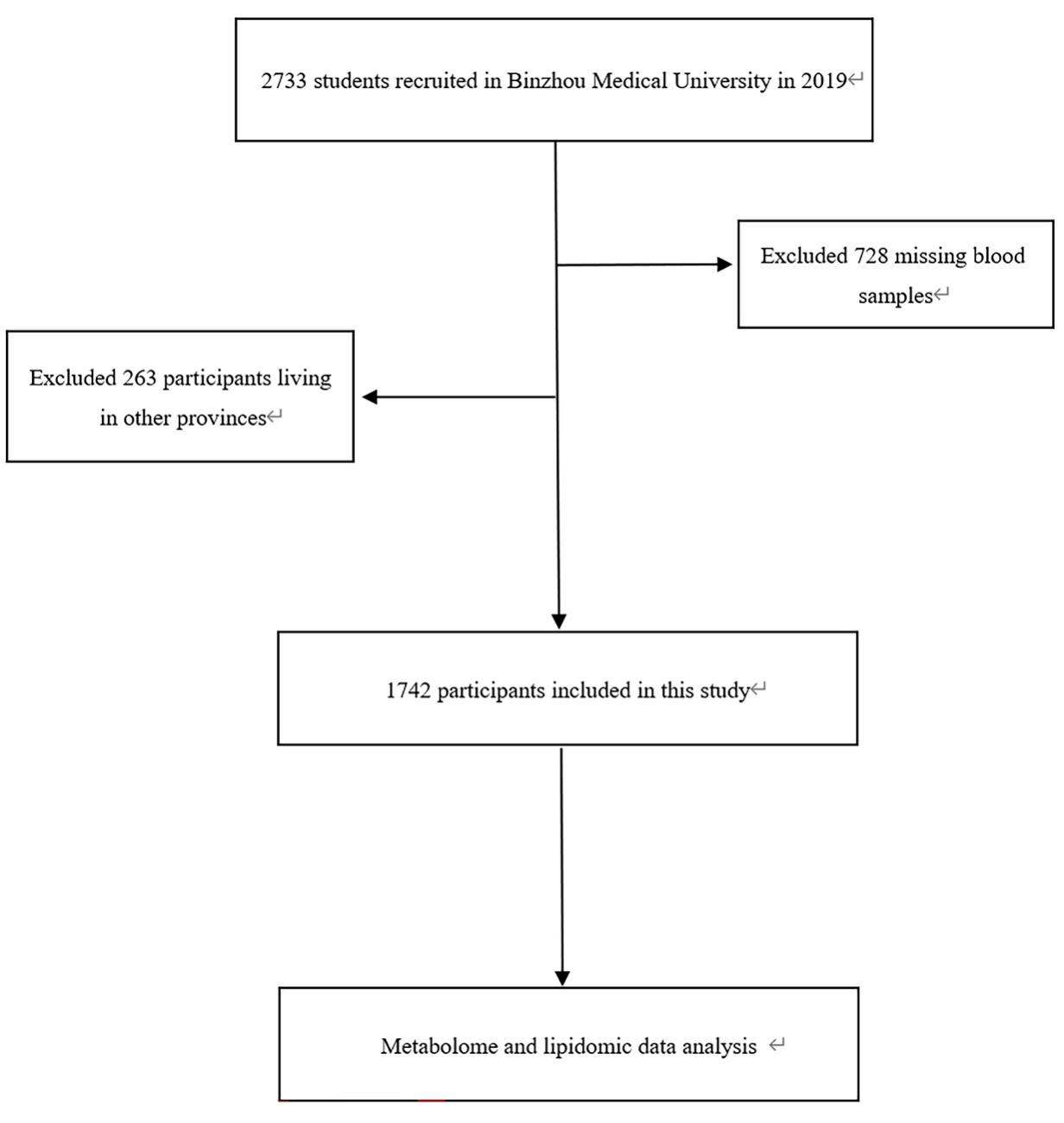


**Fig. S1.** Flow chart of participants included in the study


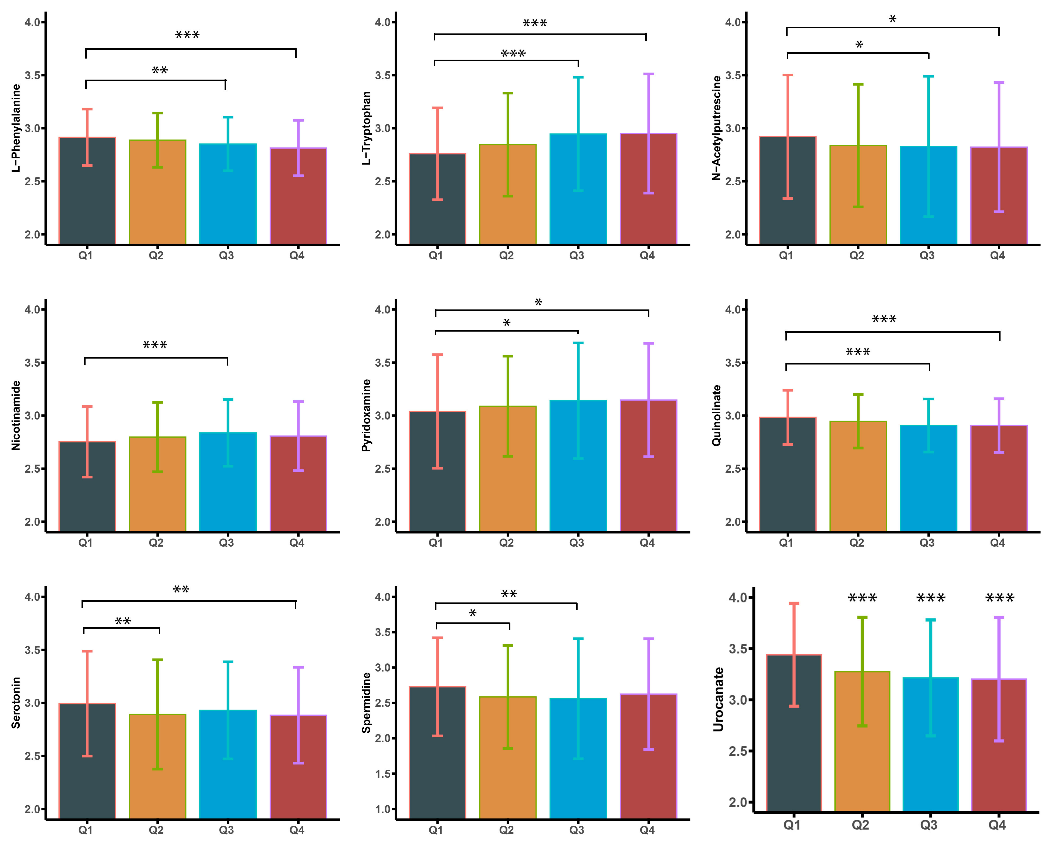


**Fig. S2.** The changes of metabolites along with the increase of blood Cu levels

Note: Q1: 25th percentile; Q2: 50th percentile; Q3: 75th percentile; Q4: 90th percentile; * represents P < 0.05, ** represents P < 0.01, ***represents P < 0.001, compared to the Q1 group.


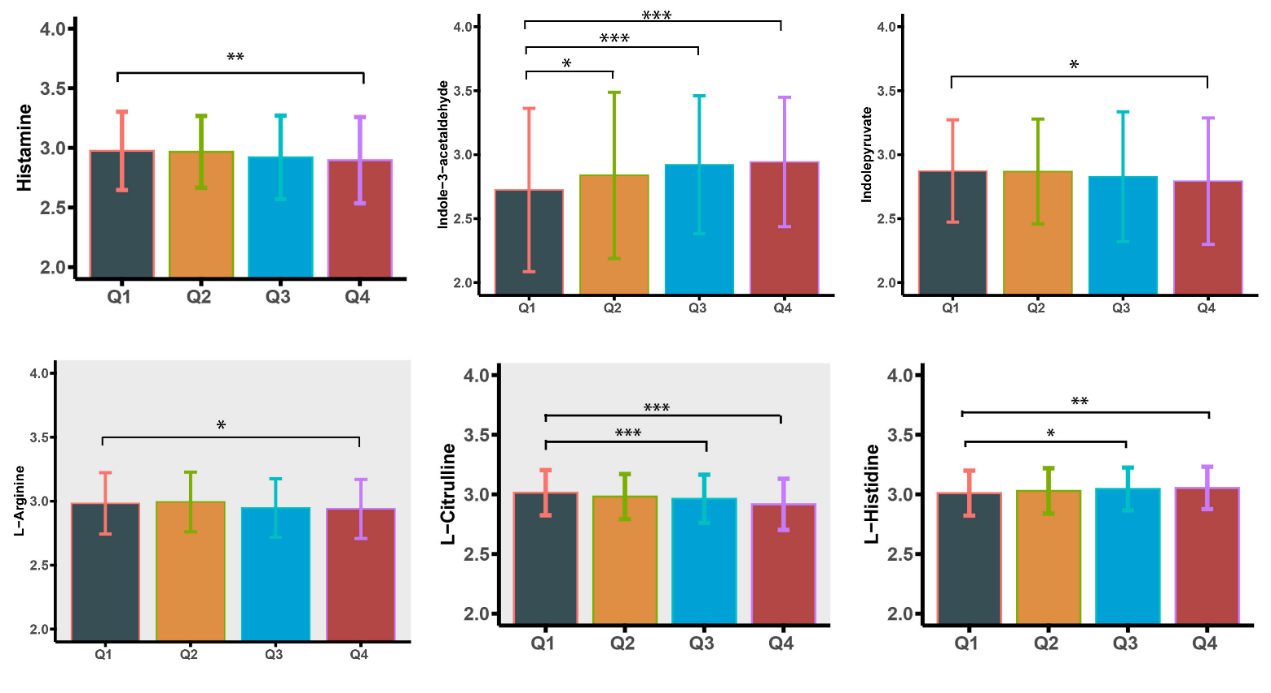


**Fig. S3.** The changes of metabolites along with the increase of blood Cu levels

Note: Q1: 25th percentile; Q2: 50th percentile; Q3: 75th percentile; Q4: 90th percentile; * represents P < 0.05, ** represents P < 0.01, ***represents P < 0.001, compared to the Q1 group.

**
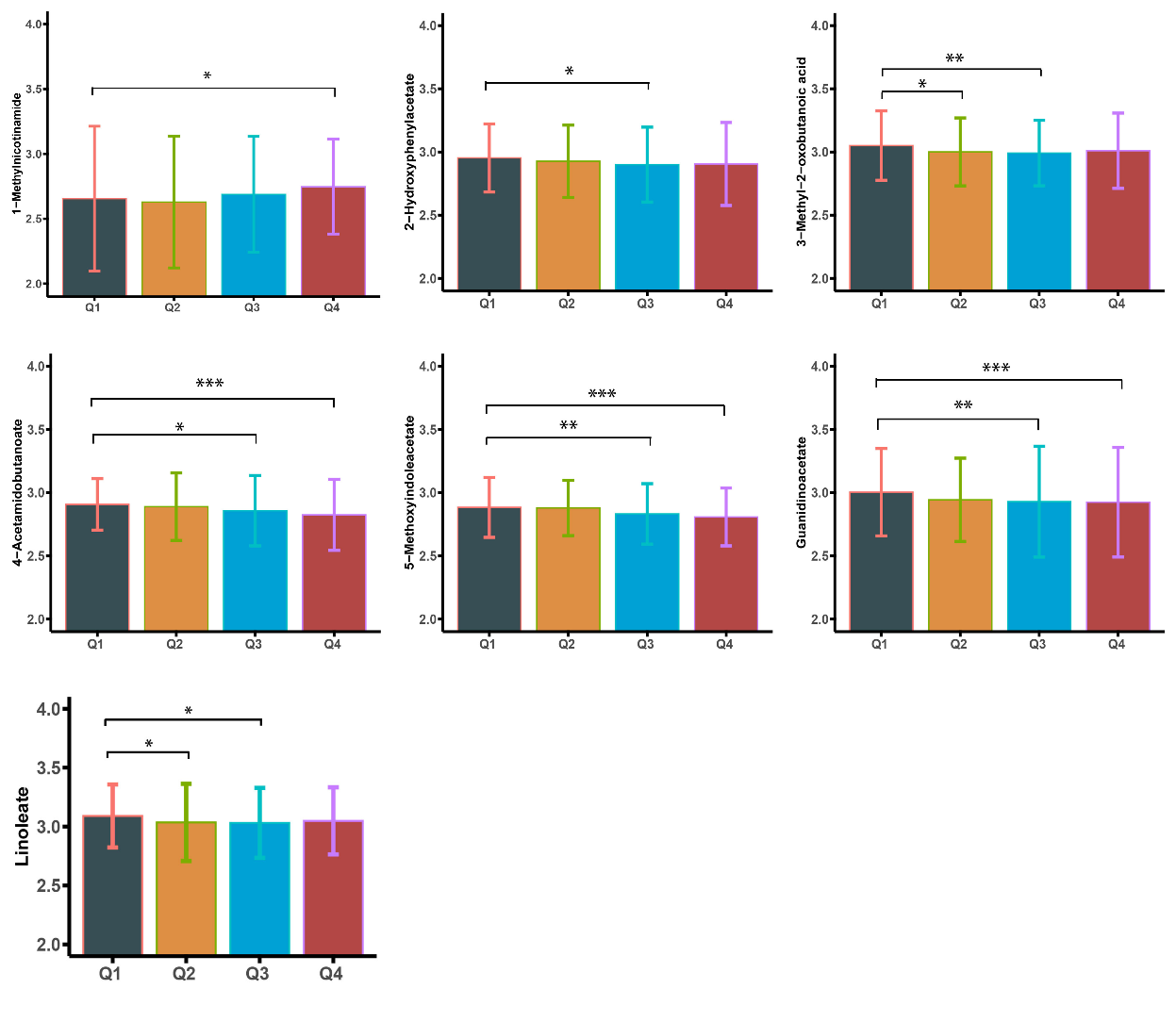
**

**Fig. S4.** The changes of metabolites along with the increase of blood Cu levels

Note: Q1: 25th percentile; Q2: 50th percentile; Q3: 75th percentile; Q4: 90th percentile; * represents P < 0.05, ** represents P < 0.01, ***represents P < 0.001, compared to the Q1 group.


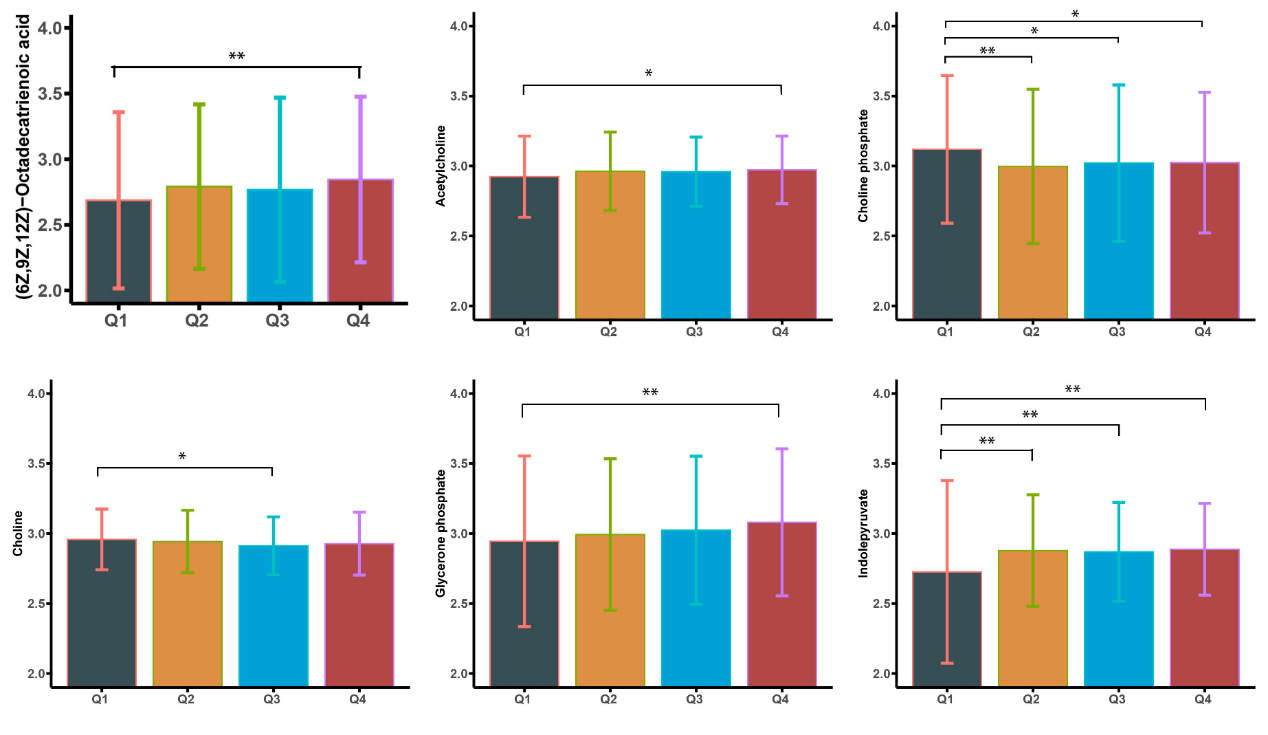


**Fig. S5.** The changes of metabolites along with the increase of blood Ce levels

Note: Q1: 25th percentile; Q2: 50th percentile; Q3: 75th percentile; Q4: 90th percentile; * represents P < 0.05, ** represents P < 0.01, ***represents P < 0.001, compared to the Q1 group.

**
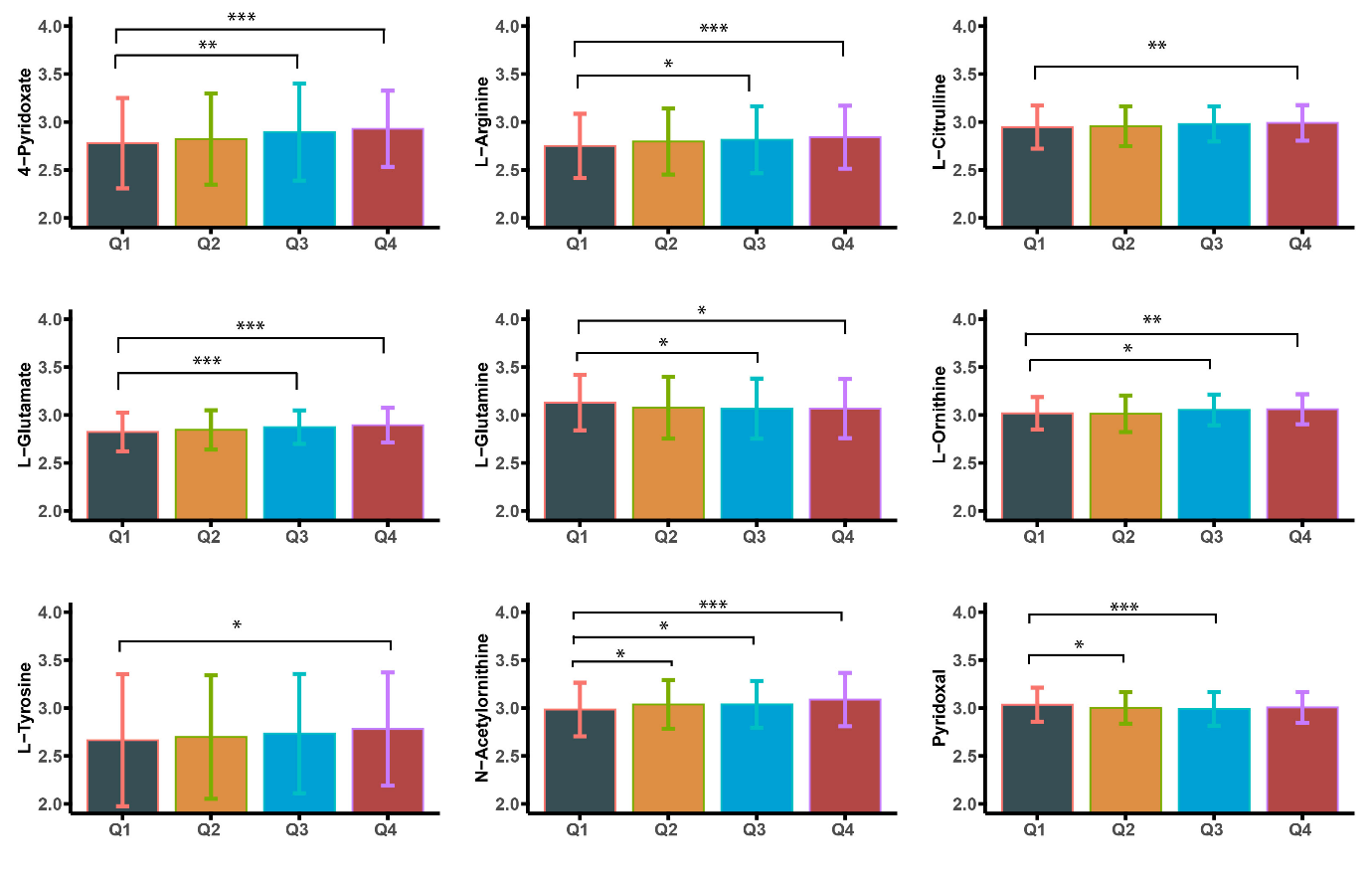
**

**Fig. S6.** The changes in metabolites with increasing blood Fe levels

Note: Q1: 25th percentile; Q2: 50th percentile; Q3: 75th percentile; Q4: 90th percentile; * represents P < 0.05, ** represents P < 0.01, ***represents P < 0.001, compared to the Q1 group.


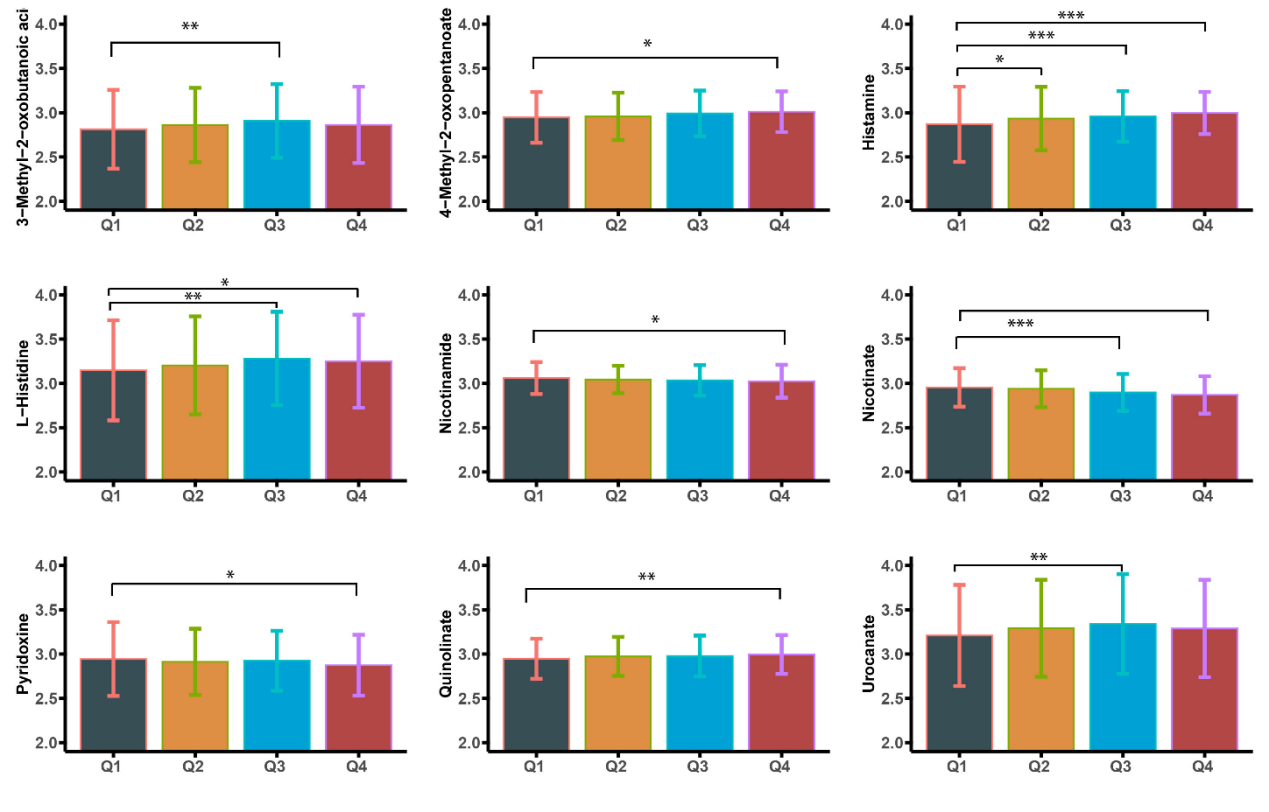


**Fig. S7.** The changes in metabolites with increasing blood Fe levels

Note: Q1: 25th percentile; Q2: 50th percentile; Q3: 75th percentile; Q4: 90th percentile; * represents P < 0.05, ** represents P < 0.01, ***represents P < 0.001, compared to the Q1 group.

**Table. S1** Descriptive statistics of metals

| **Metal** | **Statistic** | **All (n=1742)** | **Male (n=740)** | **Female (n=1002)** |
| --- | --- | --- | --- | --- |
| Ce | Geometric_Mean | 0.11 | 0.11 | 0.11 |
|  | 95%CI | 0.11－0.12 | 0.11－0.11 | 0.11－0.12 |
|  | Arithmetic_Mean | 0.16 | 0.13 | 0.19 |
|  | SD | 1.27 | 0.10 | 1.68 |
|  | Median | 0.11 | 0.11 | 0.11 |
|  | P_25_ | 0.08 | 0.08 | 0.08 |
|  | P_75_ | 0.15 | 0.14 | 0.15 |
| Cu | Geometric_Mean | 855.83 | 854.23 | 857.01 |
|  | 95%CI | 849.03－862.69 | 843.98－864.62 | 847.94－866.18 |
|  | Arithmetic_Mean | 868.49 | 866.24 | 870.15 |
|  | SD | 155.43 | 146.30 | 161.89 |
|  | Median | 852.93 | 854.07 | 852.24 |
|  | P_25_ | 765.41 | 768.90 | 764.43 |
|  | P_75_ | 953.63 | 949.18 | 955.85 |
| Fe | Geometric_Mean | 482,015.05 | 484,814.03 | 479,958.32 |
|  | 95%CI | 477029.96－487052.24 | 477301.64－492444.67 | 473317.68－486692.13 |
|  | Arithmetic_Mean | 492,712.05 | 495,213.66 | 490,864.56 |
|  | SD | 95,391.75 | 94,740.22 | 95,875.38 |
|  | Median | 500,081.92 | 503,259.48 | 495,232.59 |
|  | P_25_ | 433,110.80 | 437,181.28 | 430,892.18 |
|  | P_75_ | 558,129.93 | 561,905.96 | 555,740.30 |

**Note: Table S2-S5 were presented in Excel format due to the substantial size of the data.**

**TableS2.** Screening process for Cu-related metabolites

**Table S3.** Tukey HSD Results

**TableS4.** Screening process for Ce-related metabolites

**TableS5.** Screening process for Fe-related metabolites

**Table. S6** The changes of metabolic pathway along with the increase of blood Cu levels

| **Pathway Name** | **P** | **FDR** |
| --- | --- | --- |
| [Arginine biosynthesis](https://new.metaboanalyst.ca/Secure/pathway/PathResultView.xhtml) | <0.001 | 0.022 |
| [Phenylalanine metabolism](https://new.metaboanalyst.ca/Secure/pathway/PathResultView.xhtml) | <0.001 | 0.070 |
| [Vitamin B6 metabolism](https://new.metaboanalyst.ca/Secure/pathway/PathResultView.xhtml) | <0.001 | 0.022 |
| [Pantothenate and CoA biosynthesis](https://new.metaboanalyst.ca/Secure/pathway/PathResultView.xhtml) | <0.01 | 0.104 |
| [beta-Alanine metabolism](https://new.metaboanalyst.ca/Secure/pathway/PathResultView.xhtml) | <0.01 | 0.114 |
| [Valine, leucine and isoleucine biosynthesis](https://new.metaboanalyst.ca/Secure/pathway/PathResultView.xhtml) | <0.01 | 0.070 |
| [Biosynthesis of unsaturated fatty acids](https://new.metaboanalyst.ca/Secure/pathway/PathResultView.xhtml) | <0.01 | 0.077 |
| [Histidine metabolism](https://new.metaboanalyst.ca/Secure/pathway/PathResultView.xhtml) | <0.01 | 0.074 |
| [Pyrimidine metabolism](https://new.metaboanalyst.ca/Secure/pathway/PathResultView.xhtml) | <0.01 | 0.094 |
| [Citrate cycle (TCA cycle)](https://new.metaboanalyst.ca/Secure/pathway/PathResultView.xhtml) | <0.01 | 0.103 |
| [Phenylalanine, tyrosine and tryptophan biosynthesis](https://new.metaboanalyst.ca/Secure/pathway/PathResultView.xhtml) | 0.011 | 0.694 |
| [Arginine and proline metabolism](https://new.metaboanalyst.ca/Secure/pathway/PathResultView.xhtml) | 0.019 | 0.037 |
| [Nicotinate and nicotinamide metabolism](https://new.metaboanalyst.ca/Secure/pathway/PathResultView.xhtml) | 0.025 | 0.070 |
| [Alanine, aspartate and glutamate metabolism](https://new.metaboanalyst.ca/Secure/pathway/PathResultView.xhtml) | 0.032 | 0.261 |
| [Ubiquinone and other terpenoid-quinone biosynthesis](https://new.metaboanalyst.ca/Secure/pathway/PathResultView.xhtml) | 0.048 | 0.787 |

**Table. S7** The changes of metabolic pathway along with the increase of blood Ce levels

| **Pathway Name** | **P** | **FDR** |
| --- | --- | --- |
| [Valine, leucine and isoleucine biosynthesis](https://www.metaboanalyst.ca/Secure/pathway/PathResultView.xhtml) | 0.001 | 0.072 |
| [Vitamin B6 metabolism](https://www.metaboanalyst.ca/Secure/pathway/PathResultView.xhtml) | 0.001 | 0.072 |
| [Nicotinate and nicotinamide metabolism](https://www.metaboanalyst.ca/Secure/pathway/PathResultView.xhtml) | 0.007 | 0.171 |
| [Biosynthesis of unsaturated fatty acids](https://www.metaboanalyst.ca/Secure/pathway/PathResultView.xhtml) | 0.016 | 0.091 |
| [Taurine and hypotaurine metabolism](https://www.metaboanalyst.ca/Secure/pathway/PathResultView.xhtml) | 0.019 | 1 |
| [One carbon pool by folate](https://www.metaboanalyst.ca/Secure/pathway/PathResultView.xhtml) | 0.0329 | 1 |

**Table. S8** The changes of metabolic pathway along with the increase of blood Fe levels

| **Pathway Name** | **p** | **FDR** |
| --- | --- | --- |
| [Phenylalanine metabolism](https://www.metaboanalyst.ca/Secure/pathway/PathResultView.xhtml) | <0.001 | 0.005 |
| [Arginine biosynthesis](https://www.metaboanalyst.ca/Secure/pathway/PathResultView.xhtml) | <0.001 | <0.001 |
| [Vitamin B6 metabolism](https://www.metaboanalyst.ca/Secure/pathway/PathResultView.xhtml) | <0.001 | 0.006 |
| [Histidine metabolism](https://www.metaboanalyst.ca/Secure/pathway/PathResultView.xhtml) | <0.001 | 0.006 |
| [Nicotinate and nicotinamide metabolism](https://www.metaboanalyst.ca/Secure/pathway/PathResultView.xhtml) | 0.004 | 0.039 |
| [Biosynthesis of unsaturated fatty acids](https://www.metaboanalyst.ca/Secure/pathway/PathResultView.xhtml) | 0.005 | 0.263 |
| [Arginine and proline metabolism](https://www.metaboanalyst.ca/Secure/pathway/PathResultView.xhtml) | 0.005 | 0.263 |
| [Glutathione metabolism](https://www.metaboanalyst.ca/Secure/pathway/PathResultView.xhtml) | 0.008 | 0.198 |
| [Phenylalanine, tyrosine and tryptophan biosynthesis](https://www.metaboanalyst.ca/Secure/pathway/PathResultView.xhtml) | 0.012 | 0.107 |
| [Glyoxylate and dicarboxylate metabolism](https://www.metaboanalyst.ca/Secure/pathway/PathResultView.xhtml) | 0.014 | 0.263 |
| [Nitrogen metabolism](https://www.metaboanalyst.ca/Secure/pathway/PathResultView.xhtml) | 0.028 | 0.197 |
| [Butanoate metabolism](https://www.metaboanalyst.ca/Secure/pathway/PathResultView.xhtml) | 0.029 | 0.426 |
| [Pyrimidine metabolism](https://www.metaboanalyst.ca/Secure/pathway/PathResultView.xhtml) | 0.032 | 0.191 |
| [Alanine, aspartate and glutamate metabolism](https://www.metaboanalyst.ca/Secure/pathway/PathResultView.xhtml) | 0.038 | 0.392 |
| [Valine, leucine and isoleucine biosynthesis](https://www.metaboanalyst.ca/Secure/pathway/PathResultView.xhtml) | 0.049 | 0.042 |
